# Titanium Alloy Materials with Very High Cycle Fatigue: A Review

**DOI:** 10.3390/ma17122987

**Published:** 2024-06-18

**Authors:** Yuhang Wu, Weifeng He, Haitao Ma, Xiangfan Nie, Xiaoqing Liang, Jile Pan, Shiguang Wang, Min Shang, Li Cheng

**Affiliations:** 1 National Key Lab of Aerospace Power System and Plasma Technology, Air Force Engineering University, Xi’an 710038, China; yuhangwu106@gmail.com (Y.W.); niexiangfan_kgd@126.com (X.N.); liangxiaoqing_6366@163.com (X.L.); wangshiguang228@163.com (S.W.); 2 School of Mechanical Engineering, Xi’an Jiao Tong University, Xi’an 710049, China; 13335356500@163.com; 3 School of Materials Science and Engineering, Dalian University of Technology, Dalian 116024, China; shangxiaomin2019@mail.dlut.edu.cn

**Keywords:** titanium alloys, experimental methods, macroscopic and microscopic characteristics, fatigue model, very high cycle fatigue, crack initiation

## Abstract

As the reliability and lifespan requirements of modern equipment continues to escalate, the problems with very high cycle fatigue (VHCF) has obtained increasingly widespread attention, becoming a hot topic in fatigue research. Titanium alloys, which are the most extensively used metal materials in the modern aerospace industry, are particularly prone to VHCF issues. The present study systematically reviewed and summarized the latest (since 2010) developments in VHCF research on titanium alloy, with special focus on the (i) experimental methods, (ii) macroscopic and microscopic characteristics of the fatigue fractures, and (iii) construction of fatigue fracture models. More specifically, the review addresses the technological approaches that were used, mechanisms of fatigue crack initiation, features of the S–N curves and Goodman diagrams, and impact of various factors (such as processing, temperature, and corrosion). In addition, it elucidates the damage mechanisms, evolution, and modeling of VHCF in titanium alloys, thereby improving the understanding of VHCF patterns in titanium alloys and highlighting the current challenges in VHCF research.

## 1. Introduction

Very high cycle fatigue (VHCF) characterizes fatigue phenomena for cycles ranging from 10^7^ to 10^12^, thus highlighting an ultralong lifespan fatigue. As distinguished at the microscale level and compared with traditional high cycle fatigue (HCF), VHCF is not only a matter of an extended lifespan, which ranges from 10^5^ to 10^7^ cycles. The mechanisms of fatigue initiation and initial propagation are notably more complex and vary largely depending on the material type [1].

Systematic research on VHCF began in the 1980s. The earliest documented studies appeared in 1983 when Naito et al., from Komatsu Ltd. in Tokyo, Japan, reported on the stress–life (S–N) curve of surface-carburized SCM415 steel (15CrMo). This S–N curve was obtained using a 50 Hz rotating-bending fatigue testing machine. It showed a “stepwise” shape that indicated the absence of a traditional fatigue limit [2]. Concurrently, Atrens et al., from Brown Boveri Research Center, in Switzerland, reported on the S–N curve for a Ti6Al4V titanium alloy, which was obtained using a combination of the following three different testing machines: a 60 Hz servo-hydraulic testing machine, a 150 Hz electromagnetic resonance testing machine, and a 20 kHz ultrasonic testing machine. This S–N curve showed a “double-step” pattern, with the second step appearing after 10^9^ cycles. Also, the S–N curve for the X20CrMoV121 martensitic stainless steel showed a continuous decline, with the fatigue limit extended beyond 10^9^ cycles [3]. Both studies observed the phenomenon of fatigue crack initiation in the subsurface of the specimens after 10^7^ cycles, which has become a characteristic feature of VHCF.

Over the past four decades, VHCF research has received increasing attention and emerged as a new focus within the study of fatigue fractures. The reason for this increased interest is attributed to two main factors. Firstly, the operational speeds of modern mechanical equipment (such as in airplanes, high-speed trains, and automobiles) have significantly increased, along with a corresponding increase in lifespan requirements. This has led to mechanical components with several load cycles that exceed the traditional high-cycle fatigue (HCF) threshold. Secondly, most metallic materials do not exhibit a conventional fatigue limit after 10^7^ cycles [4], and the mechanisms of crack initiation and initial propagation differ from those observed in traditional HCFs. This necessitates the further exploration of the fatigue mechanisms of many materials.

Several review papers on VHCF have been published since 2010, which present related issues from various perspectives. Among the most comprehensive reviews are those by Pyttel (2011) [5], Zimmermann (2012) [6], Li (2012) [7], Mayer (2016) [8], Li Yong-de (2016) [9], Sakai (2016) [10], Hong You-shi (2018) [11], Costa (2020) [12], Sharma (2020) [13], Tridello (2021) (2022) [14,15], Caivano (2021) [16], Avateffazeli (2022) [17], Sakai (2023) [18], and Tusher (2023) [19]. Most of these studies focus on alloy steels. Given the relatively low proportion of alloy steel used in aircrafts, which typically involve classical models and applications, and by considering that titanium alloys are not only largely used but have also specific VHCF requirements, the present paper primarily discusses VHCF issues in aerospace titanium alloy materials. It provides a comprehensive review of the latest advancements in VHCF research on titanium alloys, with a focus on (i) experimental methods, (ii) macroscopic and microscopic characteristics, and (iii) model construction. More specifically, the review elucidates the characteristics, mechanisms, and models of the VHCF behavior in titanium alloys.

## 2. VHCF Issues for Aerospace Titanium Alloy Materials

Titanium is less dense than steel by 40%. However, its strength is comparable to that of steel, which improves structural efficiency. In addition, titanium exhibits an excellent heat resistance, corrosion resistance, elasticity, anti-elasticity, and formability [20]. It is primarily used in the following three areas of the aircraft: aero engines, airframes, and onboard equipment structures. Thus, this metal material has the largest usage in the modern aerospace industry.

Titanium alloys have completely replaced materials (such as aluminum alloys and stainless steel) in the cold-section components of advanced aero engines. Fan/compressor disks, fan/compressor blades, and casings are extensively manufactured from titanium alloys. A full-titanium scheme for compressors has even been achieved. For instance, the mass ratios of the titanium alloy reached 24% and 27% in the American third-generation F-14 and F-15 fighter jets, respectively, and it was even higher (41%) in the fourth-generation F-22 fighter jet. Also, the mass ratio of the titanium alloy was 39% in the F119 turbofan engine in the F-22 fighter jet. These examples represent the highest usage of titanium alloys in aircrafts to date. With the usage of new-generation turbofan engines in the third-generation of fighter jets, the problems with VHCF have gradually become apparent. In 1994, the United States initiated the “National Research Program on High Cycle Fatigue Science and Technology”, which did not distinguish between the HCF and VHCF concepts. The research findings indicated that the fatigue strength would continue to decline after 3 × 10^7^ cycles. The fatigue life requirement for titanium alloys should, therefore, be set at 10^9^ cycles. In 1999, the revised “Engine Structural Integrity Program” (MIL-HDBK-1783A) [21] was presented, which modified the HCF life requirements to the following: (1) 10^7^ cycles for iron-based and nickel-based superalloy parts; (2) 10^9^ cycles for titanium alloy parts; (3) 3 × 10^7^ cycles for parts made of other materials. China’s new “General Specifications for Aerospace Turbojet and Turbofan Engines” and “General Specifications for Aerospace Turboshaft and Turboprop Engines” have also set the VHCF life requirement at 10^9^ cycles for titanium alloy parts. However, since the aircraft body structures are mainly designed for low cycle fatigue, there is, currently, no standard specification for VHCF requirements globally.

In short, titanium alloy materials have become the most used metal materials in advanced aircraft and are the metals that are the most affected by VHCF issues.

## 3. Experimental Methods for VHCF

Fatigue testing is a fundamental method for obtaining material fatigue performance data and conducting fatigue research. Many fatigue testing methods are governed by international standards (such as “American Society for Testing and Materials” (ASTM), the “International Organization for Standardization” (ISO), “National Standards” (GB), and “Industry or Corporate Standards”), which standardize the procedures for using testing equipment and measurement instruments. However, because of the incomplete understanding of the mechanisms of VHCF and the initiation of cracks in the subsurface of the specimens, the impact of different testing methods (e.g., tension–compression and bending methods) on the test results has become magnified. To date, there are no specific standards or specifications for VHCF testing, which has resulted in numerous new challenges when determining whether standard fatigue testing techniques and methods should be employed or new fatigue testing technologies and methods should be developed. The earliest research [2,3] involved the following four types of fatigue testing methods: rotating-bending, electrohydraulic servo, electromagnetic resonance, and ultrasonic methods. This illustrates that the selection and application of VHCF testing methods have been a complex issue from the very beginning. Therefore, the present review categorized the testing methods as the following two main types: traditional fatigue methods and accelerated ultrasonic fatigue testing methods. These two types are discussed separately in the present review.

Traditional fatigue testing methods include rotating bending, electrohydraulic servo, electromagnetic resonance, and electromagnetic vibration tables. Most of these methods are standardized, with results that are readily accepted by the engineering community. However, the relatively low test frequencies (generally below 1000 Hz) result in VHCF tests with very long durations, making the temporal and economic costs prohibitive in the engineering sector.

**Figure 1:**
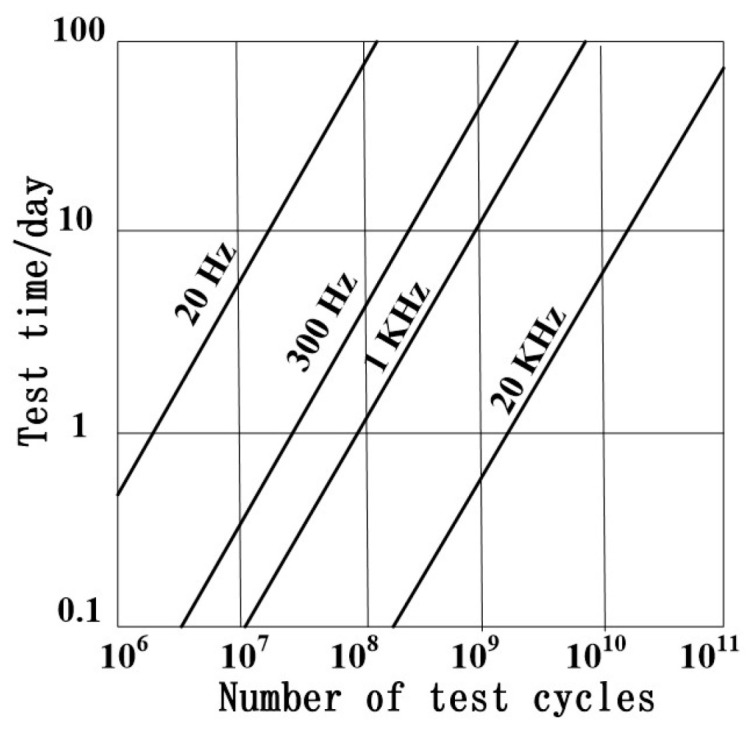
Time required for fatigue tests at different frequencies.

Ultrasonic fatigue testing is an accelerated fatigue testing method, which is based on the principle of resonance. It has a typical test frequency of 20 kHz. As can be seen in Figure 1, this method offers a significant time advantage. It largely reduces both the temporal and economic costs that are associated with fatigue testing. This has made it the most widely used method for VHCF testing to date. When the National Institute for Materials Science (NIMS), in Japan, established a fatigue database, a 10^10^ cycles fatigue test, which used a 100 Hz rotating-bending test method, required three years. On the contrary, a 20 kHz ultrasonic fatigue test required only one week. However, there is no standard for ultrasonic fatigue testing to date, which has affected the acceptance of test results among the engineering community.

In the articles included in this review, 19% used traditional testing methods, 65% used ultrasonic testing methods, and 16% combined (or compared) traditional and ultrasonic testing methods.

### 3.1. Traditional Fatigue Testing Methods

#### 3.1.1. Rotating-Bending Method

**Figure 2:**
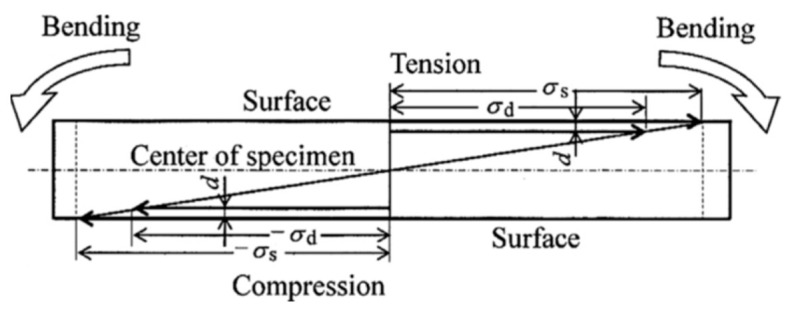
Stress distribution across the section during rotating bending [10].

**Figure 3:**
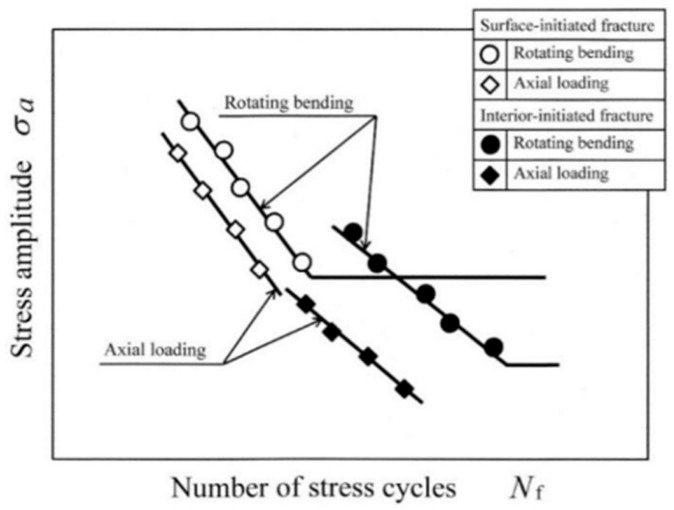
Illustration of duplex S–N characteristics for rotating bending and axial loading [10].

A rotating-bending fatigue testing machine is one of the earliest specialized fatigue testing machines. In 1860, Wohler, from Germany, used this method to study fatigue problems with train axles. The advantages with this method include its simple equipment structure, low cost, low energy consumption, reliable loading, availability of standards for reference, and the capability to test multiple specimens simultaneously. However, its drawbacks are its singular mode of loading (i.e., unable to apply a mean stress) and low testing frequency, which generally does not exceeds 100 Hz. Because of the presence of stress gradients in the specimens under rotating bending at very high cycle counts, the local stress in the crack initiation area in the subsurface is always slightly lower than the nominal bending stress at the surface (Figure 2). In other words, the stress at the surface crack initiation site during HCF is always slightly higher than the stress at the subsurface crack initiation site during VHCF. Therefore, compared to the extension line of the S–N curve for the surface crack initiation, the fatigue life of the internal crack initiation has shown an increasing trend (Figure 3). Also, S–N curves obtained by the rotating-bending method have been found to have larger impacts on the VHCF region than the S–N curve obtained by the axial loading method [10].

Since 1978, NIMS in Japan has been using the rotating-bending test method to build a fatigue database for titanium alloy materials. They completed the VHCF database in 2019 for Ti-6Al-4V titanium alloys with 900 MPa and 1100 MPa grades [22]. In 2018, Uematsu et al. [23] used a rotating-bending testing machine with a test frequency of 53 Hz to investigate the effect of ultrasonic impact treatment on the fatigue properties of β-type titanium alloys.

#### 3.1.2. Hydraulic Servo Method

The hydraulic servo fatigue testing system was developed in the early 1960s with the advent of electrohydraulic servo components. Tension–compression was the primary mode of specimen loading, and it has since then become the most commonly used standard method for material fatigue tests. The advantages with this method include high reliability, good precision, easy control of load, and the availability of standards for reference. Currently, the loading frequency can reach up to 1000 Hz, while significant errors during high-frequency operation are the main drawback.

**Figure 4:**
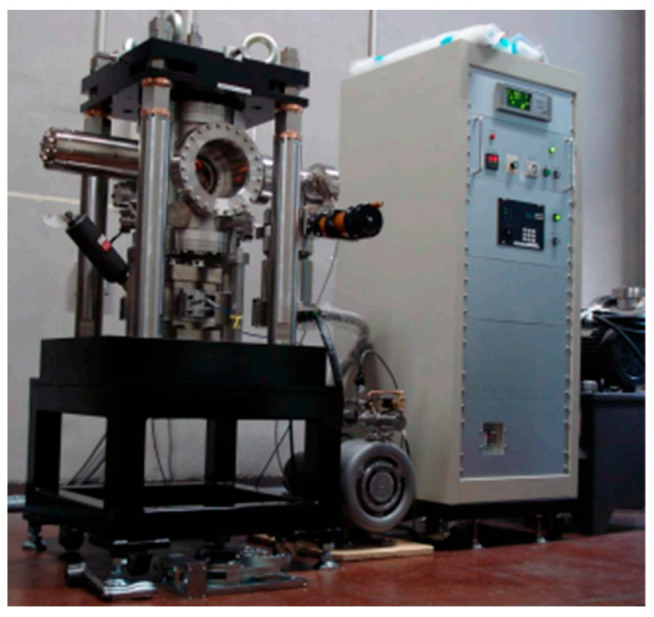
Photo of the ultrahigh vacuum fatigue testing machine [24,25,26].

In 2010 and 2014, Oguma et al. [24,25,26] developed a hydraulic servo testing system, which was equipped with an ultrahigh vacuum environment (Figure 4). It was used to study the fatigue crack propagation characteristics of Ti-6Al-4V titanium alloy in an ultrahigh vacuum environment, with a testing frequency of 60 Hz. In 2017, Jiao et al. [27] conducted VHCF tests on Ti-6Al-4V titanium alloy at different loading frequencies. They used a material test system (MTS) to investigate the effects of different loading frequencies on the fatigue properties of Ti-6Al-4V titanium alloy. The MTS was a hydraulic servo fatigue testing system with a test frequency of 20 Hz. In 2019, Fumiyoshi et al. [28] used a portable servo fatigue testing machine with a test frequency of 400 Hz to study the growth of internal fatigue cracks and the initiation and propagation of small internal fatigue cracks in Ti-6Al-4V titanium alloy.

In 2023, Li et al. [29] investigated the HCF and VHCF of TC17 titanium alloy. More precisely, they studied the effect of the stress ratio using the hydraulic servo method and conventional frequencies ranging from 20 Hz to 80 Hz.

#### 3.1.3. Electromagnetic Resonance Method

Electromagnetic resonance fatigue testing equipment, also known as high-frequency fatigue testing equipment, is a type of electromagnetic excitation resonance fatigue testing machine. Compared with a hydraulic servo fatigue testing machine, its advantages include a lower cost and higher testing frequencies, which can reach several hundreds of hertz. However, its drawbacks are lower control precision and stability.

In 2016, Huang et al. [30] used an electromagnetic resonance fatigue testing machine with a test frequency of 110 Hz to study the effects of different stress ratios on the VHCF properties of TC-17 titanium alloy. In 2017 and 2018, Li et al. [31,32] used the same technique to study the mechanism of the VHCF failure in the TC4 titanium alloy for different stress ratios. In 2019 and 2021, they further explored VHCF issues with TC11 titanium alloy [33,34], with a test frequency of 100 Hz. In 2018, Wu et al. [35] also used an electromagnetic resonance fatigue testing machine to study the effects of different stress ratios on the HCF properties of dual-phase Ti-10V-2Fe-3Al alloy. The test frequency was 110 Hz. Moreover, in 2019, Sajadifara et al. [36] used a RUMUL GIGAFORTE resonance testing machine with a test frequency of 1000 Hz to study the impact of grain size on the VHCF behavior and notch sensitivity of titanium alloys.

#### 3.1.4. Electromagnetic Vibration Table Method

**Figure 5:**
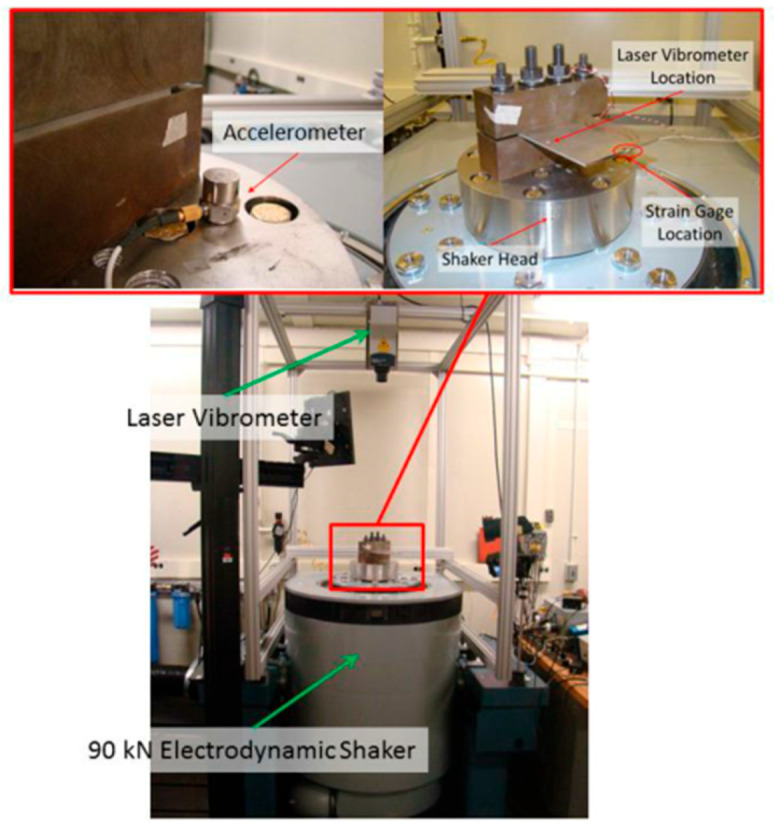
Photos of the vibration-based test setup with instrumentation [37].

The working principle of the electromagnetic vibration table method involves the alignment of the natural frequency of a specimen with the excitation frequency of the test system. A resonance is, thereby, induced that efficiently completes the test. The advantages with this method include a high loading precision and a wide range, with test frequencies up to 3 kHz. It shows good versatility and is suitable for testing both samples and actual parts. However, the design of specimens and fixtures is relatively complex and not easily mastered. In 2014, Onome et al. [37] introduced a new VHCF testing system that, by increasing the output frequency of the power supply and optimizing the geometric design of the specimen, achieved bending vibration frequencies up to 13.8 kHz. It was then possible to significantly reduce the experimental time cost (Figure 5).

**Figure 6:**
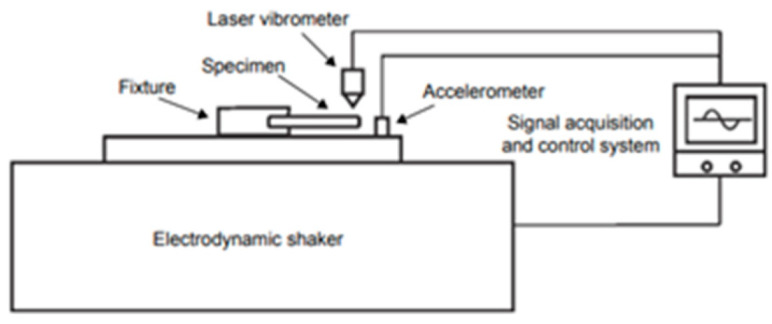
Schematic diagram of VHCF testing based on the electromagnetic vibration table [38].

**Figure 7:**
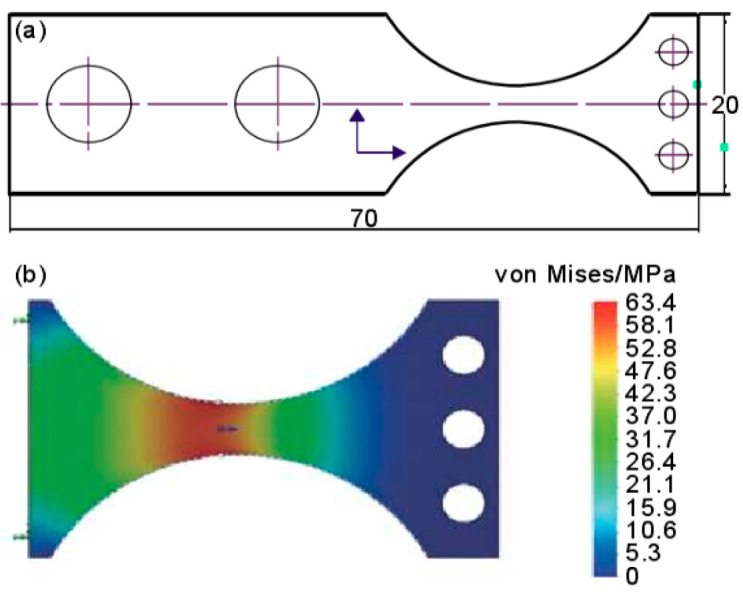
Schematic of the ultrahigh-cycle fatigue specimen [38].

In 2019, Xu et al. [38] proposed a method for VHCF tests of materials, which was based on an electromagnetic vibration table (Figure 6). They designed a TA11 titanium alloy specimen that could reach an actual loading frequency of 1756 Hz (Figure 7).

### 3.2. Ultrasonic Fatigue Testing Method

The ultrasonic fatigue testing method is also based on the principle of resonance. Its fundamental principle involves the conversion of electrical signals into required high-frequency mechanical vibration signals using a transducer, with the testing frequency typically being 20 kHz. The advantage with this method is its speed, which can significantly reduce the testing time. However, the disadvantages include the complexity of the specimen design and the specimen heating, which is caused by the high testing frequency. To apply different loading conditions on actual components, ultrasonic fatigue testing machines can use a variety of load types (including tension–compression, torsion, bending, and fretting fatigue) for different stress ratios. In 2016, Mayer reviewed the use of ultrasonic fatigue equipment with tension–compression and torsional loading modes [8].

#### 3.2.1. Tension–Compression Loading Method

**Figure 8:**
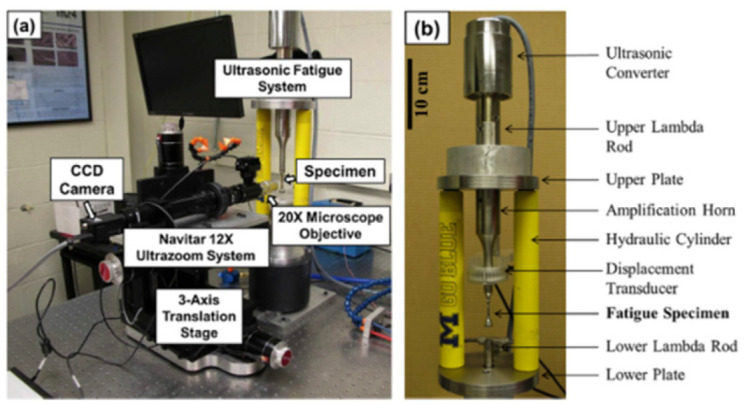
Photos of the Navitar 12X Ultrazoom optical system and ultrasonic testing system [39].

The tension–compression loading method is a classical material fatigue test loading approach, which facilitates a comparison with results from hydraulic servo fatigue tests. This method typically uses “hourglass”-, or “dog-bone”-, shaped specimens. In 2015, Geathers et al. [39] used the tension test method (Figure 8) to study the effect of the environment on the microcrack propagation in “dog-bone”-shaped specimens of Ti-6242S.

In 2017, Kasahara et al. [40] studied the VHCF properties of the β-titanium alloy Ti-22V-4Al using the tension–compression loading method. In 2017 and 2019, Chen et al. [41,42] performed VHCF tests on “dog-bone”-shaped specimens of TC4 titanium alloy. Also, in 2023, Li et al. [43] performed VHCF tests at room temperature and at 350 °C for R = −1 on laser additive-manufactured TC4 titanium alloy. They discussed the effects of additive direction, temperature, and defect size on fatigue properties.

#### 3.2.2. Torsional Loading Method

**Figure 9:**
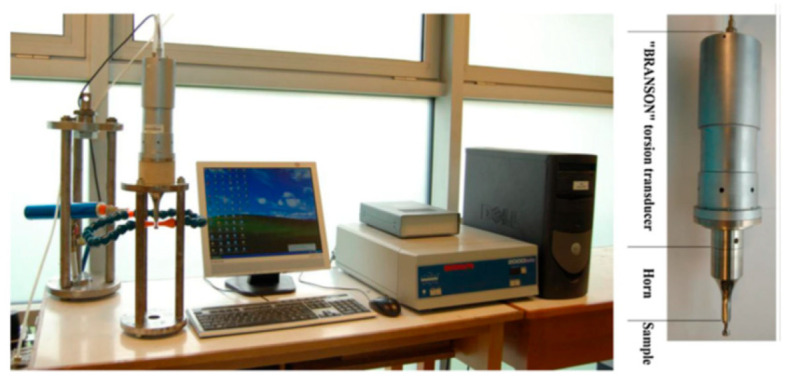
Photos of the new ultrasonic torsion testing system [42].

In 2015, Nikitin et al. [44] developed a successful ultrasonic torsional fatigue testing machine by converting excitation electrical signals into torsional vibrations of the same frequency, which was based on the torsional resonance mode of the specimen. They used a piezoelectric torsional transducer for this conversion, and the maximum torsional angle reached 0.25 mrad (Figure 9).

#### 3.2.3. Bending Loading Method

**Figure 10:**
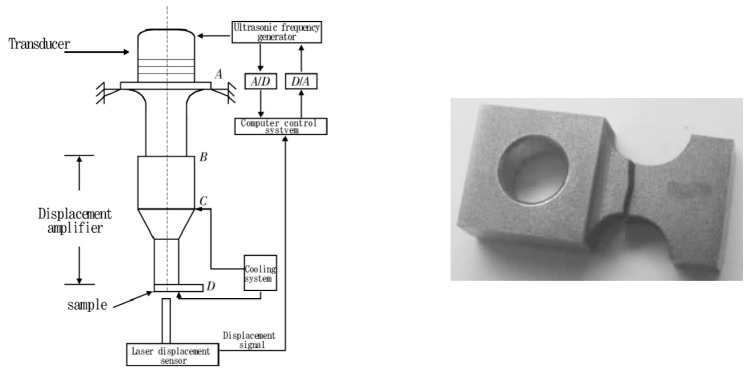
Schematic of the bending vibration ultrasonic fatigue system [45,46].

In 2012, Li et al. [45] and Gao et al. [46] constructed a cantilever beam-type ultrasonic fatigue testing system for bending vibrations at 20 kHz (Figure 10). They recognized that the primary mode of failure for aero engine blades is the fatigue fracture caused by bending vibrations.

**Figure 11:**
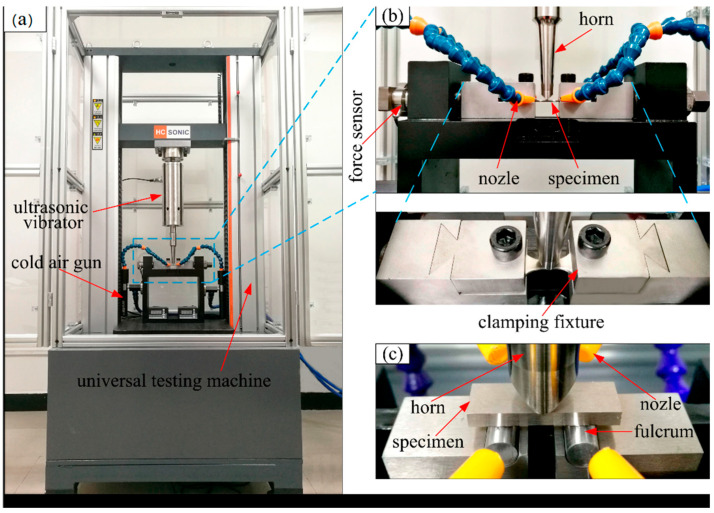
Photos of the ultrahigh-cycle fatigue bending test system with axial tension [48].

Based on an axial tension–compression ultrasonic fatigue machine, Lu et al. [47] developed, in 2019, an ultrasonic fatigue testing system, which is suitable for three-point bending tests on TC4 titanium alloy aerospace materials. They could then successfully perform ultrasonic bending fatigue tests at room temperature. Moreover, aimed at a more accurate simulation of the stress conditions of aero engine blades, Bao et al. [48] designed, in 2020, a three-point bending VHCF testing device with axial tension (Figure 11), which can be used to study the bending vibration-induced VHCF fracture mechanism in blade materials under centrifugal loads (i.e., axial tension).

In 2022, Wang et al. [49] investigated issues of VHCF fracture in aero engine compressor blades under actual working conditions. They studied the fatigue failure behavior of TC4 titanium alloy at a three-point bending–axial tension composite loading and for three different forging temperatures.

### 3.3. Fatigue Process Monitoring Methods

Real-time observations, or detections, of the initiation and propagation evolution of fatigue cracks are crucial for revealing the mechanisms of the VHCF initiation. From the available literature, the current methods for real-time monitoring of fatigue cracks mainly include synchrotron radiation imaging, electron backscatter diffraction (EBSD), and infrared thermal imaging.

#### 3.3.1. Synchrotron Radiation Imaging

**Figure 12:**
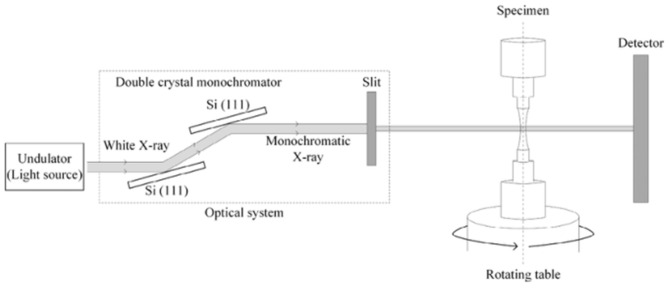
Schematic diagram of the ICT imaging system [28].

The synchrotron radiation imaging technology utilizes high-energy synchrotron radiation sources in capturing dynamic images of the fatigue crack evolution process at the atomic level (including growth mechanisms, phase transformation processes, and crack diffusion). Common imaging methods include computed tomography (CT), nano-CT, and scanning transmission X-ray microscopy (STXM). In 2016 and 2019, Yoshinaka et al. [28] used synchrotron radiation microcomputer tomography (ICT) to observe the internal crack propagation process in Ti-6Al-4V titanium alloy. A schematic of the imaging system is presented in Figure 12.

**Figure 13:**
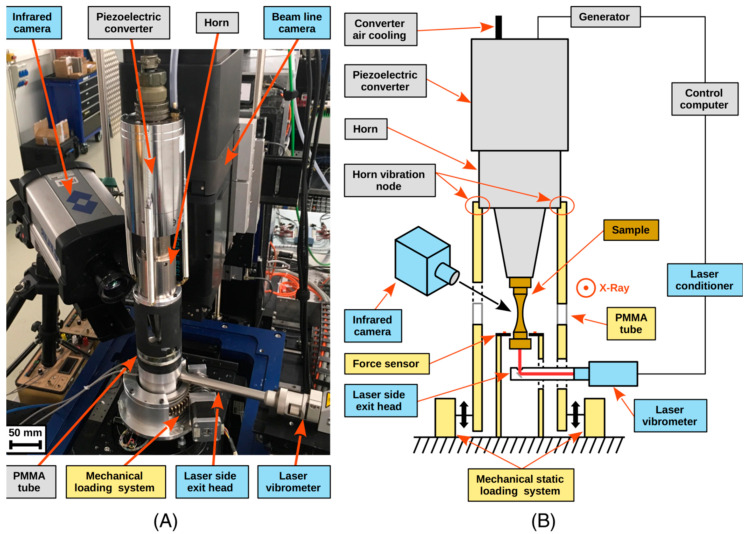
A photo of the imaging system (**A**) and Schematic diagram demonstrating the construction of the fatigue testing system (**B**) [50].

In 2020, Alexandre et al. [50] installed an X-ray tomography imaging device on an ultrasonic fatigue testing machine (Figure 13). This new testing machine can be used to study the initiation and expansion mechanisms of fatigue cracks at the microstructural end within the interior of the material.

In 2023, Hebrard et al. [51] used in situ X-ray microtomography (i.e., X-ray micro-computed tomography (μCT)) to investigate the effects of the environment (i.e., air and vacuum) on the fatigue crack propagation in a Ti64 alloy. In the same year, Fumiyoshi et al. [52] studied the initiation process of internal cracks in the VHCF (i.e., the development of internal cracks over time) in Ti-6Al-4V alloy using synchrotron radiation computed tomography (SR-CT). They proposed a possible mechanism for the formation of polycrystalline faceted clusters around the internal cracks. Also, in 2023, Tusher et al. [53] studied VHCF in additively manufactured TC4 under MC and MC-SR conditions. They performed 3D characterizations using μ-CT and introduced a new positional parameter, L, to describe the defect location types.

#### 3.3.2. EBSD Technique

**Figure 14:**
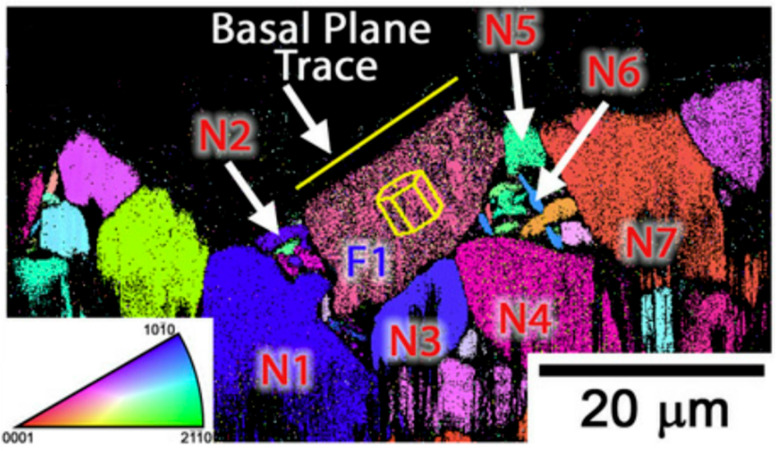
IPF graph obtained from an analysis of the FIB part by EBSD [54].

The main feature of the EBSD technique is its ability to provide submicron spatial resolution observations while retaining the conventional characteristics of a scanning electron microscope. It is then possible to obtain a large amount of crystallographic information about the sample in a very short period of time (such as grain size and shape distribution in addition to the structural properties of the grain boundaries, subgrains, twin boundaries, etc.). In 2012, Sushant et al. [54] used EBSD to analyze the orientation and slip modes of individual grains, and their neighboring grains, in a cross-section of Ti-6Al-4V titanium alloy (Figure 14).

In 2018, Uematsu et al. [23] observed VHCF cracks in Ti-22V-4V titanium alloy and attributed the initiation of interior cracks to the heterogeneity of the microstructure.

#### 3.3.3. Infrared Thermal Imaging Technique

**Figure 15:**
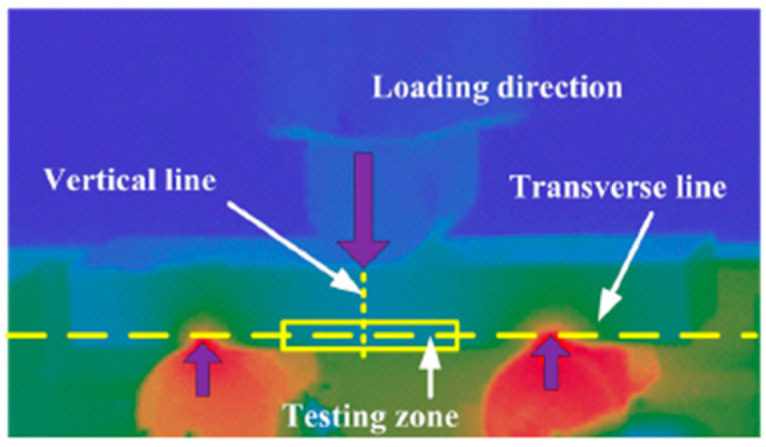
Resulting image from the infrared temperature analysis [47].

Infrared thermal imaging is an efficient technique for the real-time monitoring of surface temperature changes in specimens. It primarily uses an infrared thermal imager to measure the heat map, which is generated by the difference in infrared radiation between the target and its background. In 2019, Lu et al. [47] used the infrared thermal imaging technique to study the heat generated during the ultrasonic fatigue testing process with three-point bending on TC4 titanium alloy and composite materials (Figure 15).

## 4. Macroscopic and Microscopic Characteristics of the VHCF

Over the past four decades, research on VHCF has primarily used phenomenological analysis methods based on experimental results. This section describes the macroscopic characteristics of the VHCF, which are based on statistical curves from experimental results (such as S–N curves and Goodman diagrams), in addition to the microscopic characteristics of crack initiation and initial propagation, which are based on fracture morphology.

### 4.1. S–N Curves

#### 4.1.1. Acquisition and Basic Characteristics of S–N Curves

**Figure 16:**
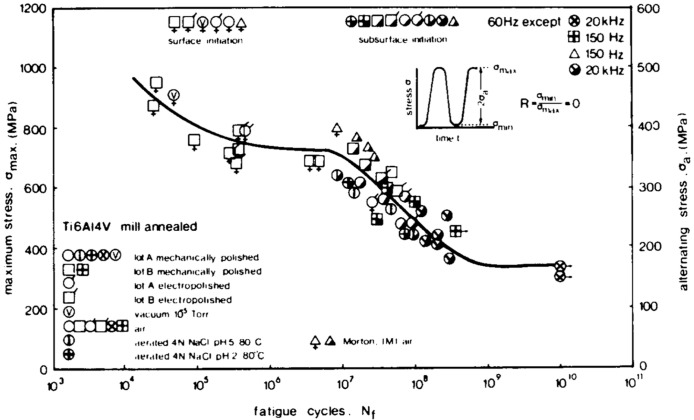
Fatigue curve distribution for milling-annealed Ti-6A1-4V [3].

For a low-cycle or high-cycle fatigue range, the obtained S–N curve of a material has a standardized basis. However, for the VHCF range, researchers have both scientifically and economically explored experimental methods for many years, which is due to the lack of standard references. In the earliest research literature, S–N curves were obtained by integrating traditional methods (in the range of 5 × 10^4^ cycles–5 × 10^8^ cycles) with ultrasonic methods (in the range of 10^7^ cycles–10^10^ cycles) (Figure 16) [3]. This literature established the basic technical route for the exploration of titanium alloy fatigue issues. Standard traditional methods have mainly been used in the HCF range, and ultrasonic methods have primarily been used in the VHCF range. Also, both traditional and ultrasonic methods have been used in the range of 10^7^ cycles–10^8^ cycles.

International standards for S–N curves have only a single plateau, which corresponds to the fatigue limit of the material. It is characterized as a “single plateau with limit” [55]. For ease of description, this review has categorized the S–N curves of titanium alloys into four types, which are based on the characteristics found in the reviewed literature: “single plateau with limit”, “no plateau, no limit”, “single plateau without limit”, and “dual plateaus with limits”.

Because of the accelerated nature of ultrasonic testing methods, the engineering community often scrutinizes obtained results. The following conclusions were drawn from extensive comparative studies: Both large cycle numbers and high load ratios promote the initiation of internal cracks in the specimens. Test frequency has a small effect on lifespan if cracks initiate in the interior of the materials. On the other hand, some studies report higher cyclic strengths at ultrasonic frequencies if the cracks initiate on the surface of the specimens. This may be due to the strain rate effect or the humidity environmental effect. Therefore, further research is needed to better understand the frequency effect on titanium alloys [8]. Furthermore, even though fatigue cracks usually initiate on the surface, an interior initiation takes place in Ti-6Al-4V titanium alloys when the fatigue limit disappears. Also, the results from ultrasonic fatigue tests have been found to align well with conventional fatigue tests [23] for the interior initiation of fatigue cracks.

**Figure 17:**
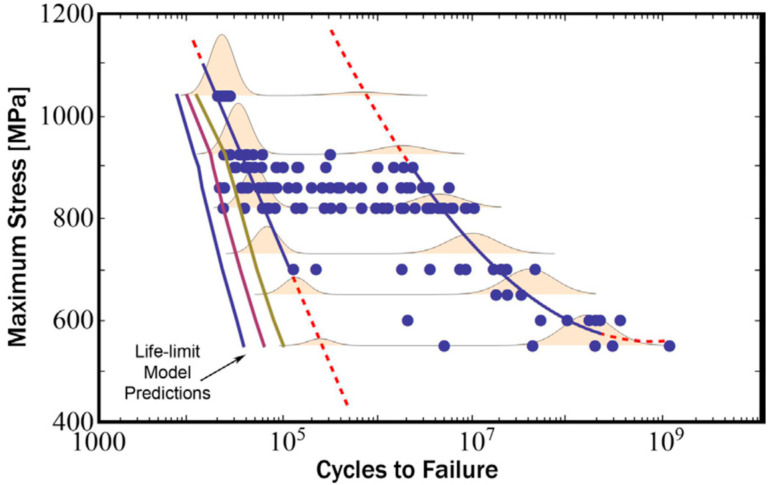
Schematic diagram showing the results of the regression analysis, which is based on the fatigue life [56]. (i) individual regression lines (blue and dashed) to Type I and Type II data, (ii) schematic bimodal probability distribution transitioning from Type I to Type II dominance with decreasing stress, and (iii) Monte Carlo predictions of 50%, 10%, and 0.1% probabilities of failure for the minimum lifetimes.

Different experimental methods result in different stress distributions within the sample, thereby yielding different S–N curves. In 2013, Larsen et al. [56] found that the tension–compression load-generated S–N curve of a Ti-6246 titanium alloy, which was obtained by a combination of traditional and ultrasonic methods, was of the type “single plateau without limit” (Figure 17). The test frequency for the portion above 750 MPa was 20 Hz, and the test frequency was 20 kHz for the portion below 750 MPa.

**Figure 18:**
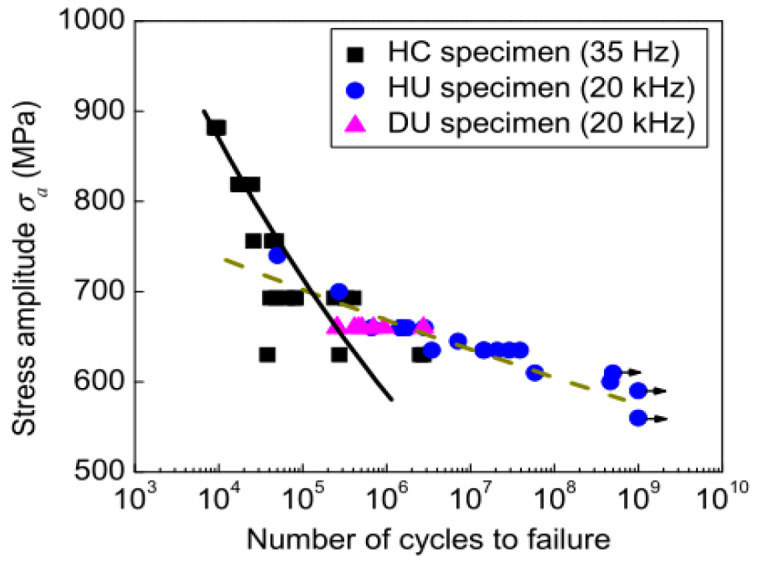
S–N fatigue life distributions of specimens with different geometries [57].

In 2018, Li et al. [57] compared the VHCF properties of Ti-6Al-2Sn-2Zr-3Mo-X titanium alloy under tension–compression loads using hydraulic servo and ultrasonic methods. It was found that the S–N curves had a “no plateau, no limit” pattern at both frequencies (Figure 18).

**Figure 19:**
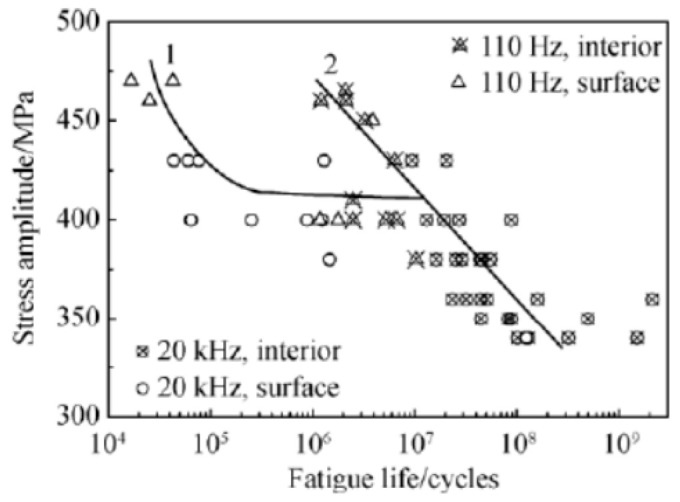
Fatigue life distribution of TC17 titanium alloy [58].

In the same year, Liu et al. [58] obtained S–N curves for TC17 titanium alloy under sinusoidal asymmetric tension–compression loads using 110 Hz electromagnetic resonance and 20 kHz ultrasonic methods. They showed a “single plateau without limit” pattern in the VHCF range, as illustrated in Figure 19.

**Figure 20:**
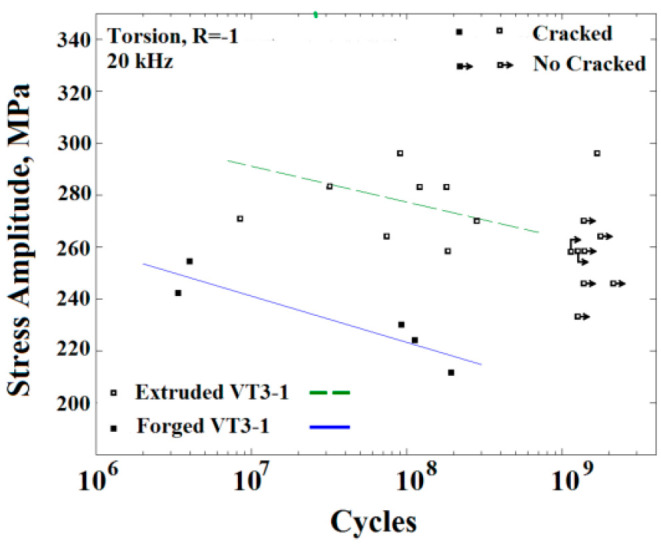
Fatigue data for VT3-1 titanium alloy after forging and extrusion treatments [59].

In 2014, Bathias et al. [59] obtained a torsional load S–N curve for VT3-1 titanium alloy using a newly developed ultrasonic fatigue testing machine that operated under pure torsion (Figure 20). It showed a “no plateau, no limit” condition.

**Figure 21:**
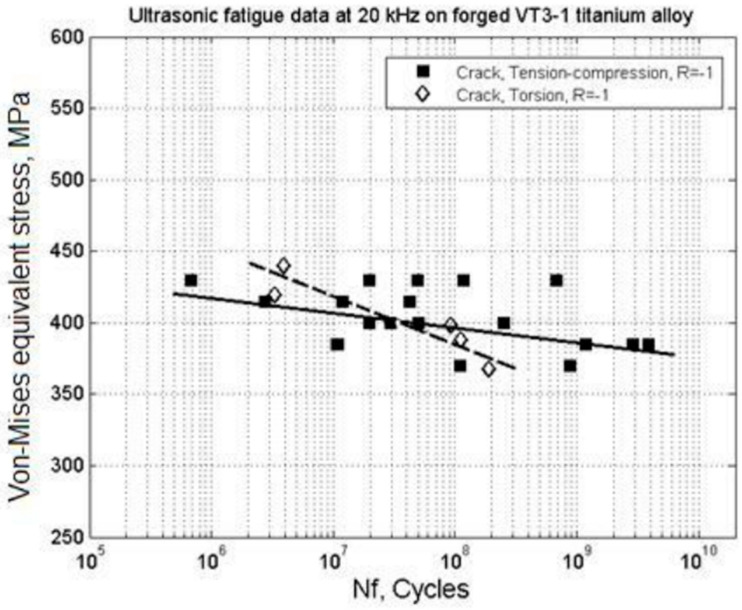
S–N curves for VT3-1 titanium alloy under combined axial and torsional loads [60].

In 2016, Nikitina et al. [60] used the ultrasonic method to obtain S–N curves for a VT3-1 titanium alloy under axial and torsional composite loads, both showing a “no plateau, no limit” pattern (Figure 21). The slope of the S–N curve for the ultrasonic torsional load was steeper than the slope for the tensile load.

**Figure 22:**
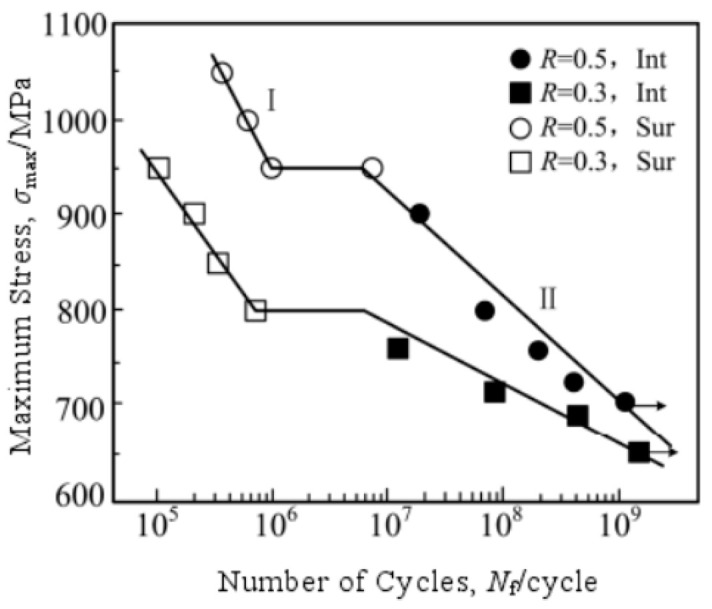
S–N curves of TC4 titanium alloy for different stress ratios [47].

In 2016, Jiao et al. [61] performed vibration bending fatigue tests on Ti-6Al-4V titanium alloy using a 300 Hz vibration table method and a 20 kHz ultrasonic method. They found that the high-frequency loading reduced the fatigue mechanical properties of the material. In 2019, Lu et al. [47] performed three-point bending ultrasonic fatigue tests on a TC4 titanium alloy, and they obtained an S–N curve with a “single plateau without limit” pattern (Figure 22).

#### 4.1.2. Influence of Specimen Condition on the S–N Curve

**Figure 23:**
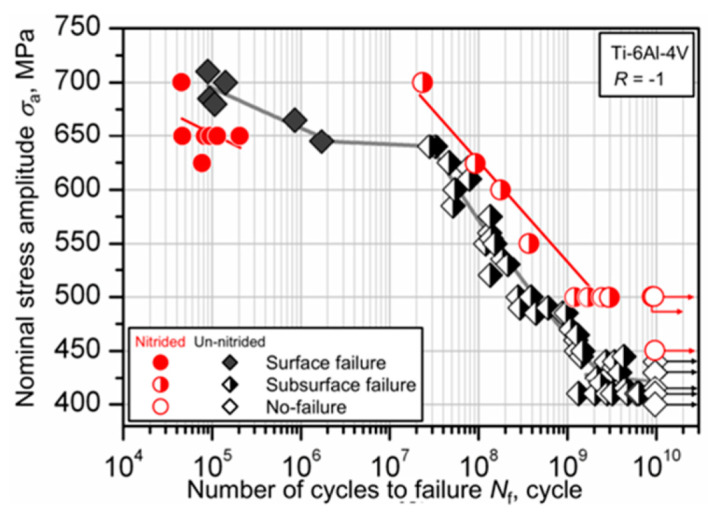
As a result of constant amplitude tests, S–N characteristics for non-nitrided and nitrided specimens [63].

The condition of the specimen is a critical internal factor that determines the VHCF performance of the material. The impact on the fatigue performance has been studied from various aspects, such as microstructural state, surface processing state, and damage state of the specimen. In 2017, Vincenzo et al. [62] used an ultrasonic fatigue equipment to study the distribution characteristics of VHCF S–N curves for Ti-6Al-4V with different microstructures. They found that the S–N curves for both bimodal and “basketweave” microstructures showed a “no plateau, no limit” pattern. In 2016, Kikuchi et al. [63] found that the fatigue S–N curve of Ti-6Al-4V changed from a “single plateau without limit” to a “no plateau, no limit” pattern after nitriding treatment (Figure 23). This indicated that a low-temperature nitriding treatment will reduce the surface fatigue life of titanium alloys.

**Figure 24:**
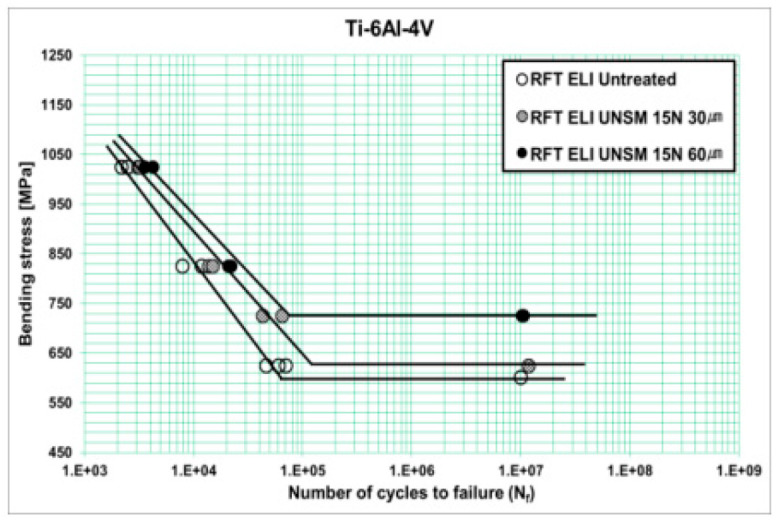
S–N curve for Ti-6Al-4V ELI after UNSM treatment [64].

By using a rotating-bending fatigue equipment, Pyun et al. [64] studied in 2014 the variations in the fatigue S–N curves for a Ti-6Al-4V titanium alloy after ultrasonic nanocrystalline surface modification (UNSM). The UNSM is a new surface treatment technology. They found that the curve had a “single plateau with limit” pattern (Figure 24). In addition, the UNSM resulted in a delayed fatigue crack growth, with an increased fatigue strength of the ELI alloy by 21% at 10^7^ cycles.

**Figure 25:**
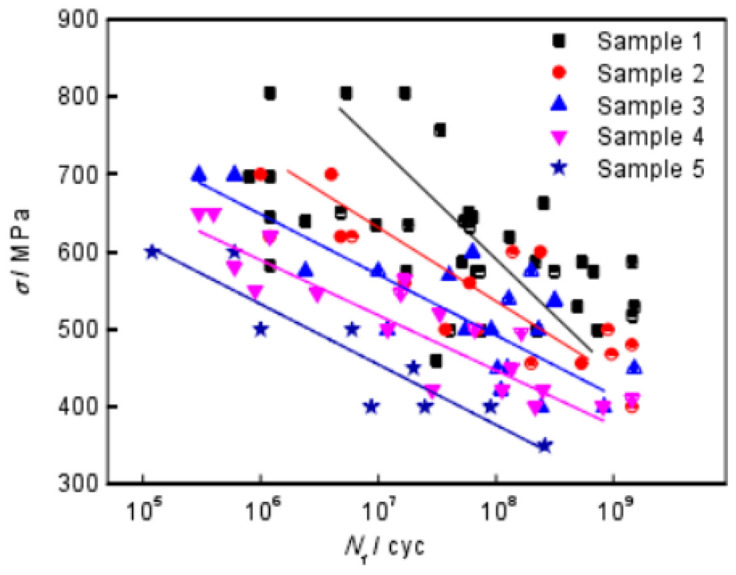
Fatigue life of Ti-6Al-4V alloy for different surface roughnesses [65].

In 2016, Zhu et al. [65] studied the impact of surface roughness on the fatigue life of titanium alloys. The S–N curves for the Ti-6Al-4V titanium alloy, which were obtained at different surface roughness levels, were all with a “no plateau, no limit” pattern (Figure 25). When the depth of the surface indentation exceeded a critical value, the fatigue life of Ti-6Al-4V alloy decreased with an increase in surface roughness. In addition, with an increase in cycle numbers, the sensitivity of the fatigue performance-to-surface roughness decreased.

**Figure 26:**
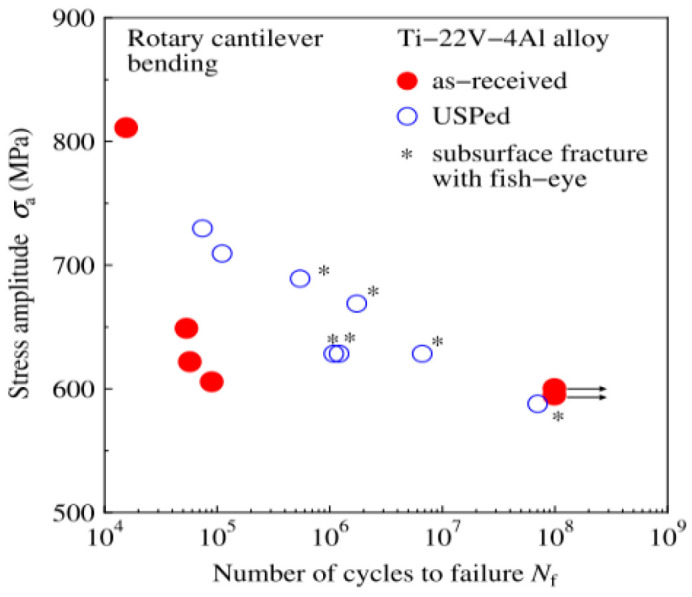
Effect of USP on the fatigue life of Ti-22V-4Al [23].

In 2018, Uematsu et al. [23] investigated the effect of the ultrasonic shot peening (USP) surface treatment on the fatigue performance of beta titanium alloys. They found that the fatigue S–N curve had a “no plateau, no limit” pattern for a rotating-bending load (Figure 26). The USP could enhance the fatigue strength in the finite life region (10^5^ cycles–10^7^ cycles), but its impact in the VHCF region was not significant.

**Figure 27:**
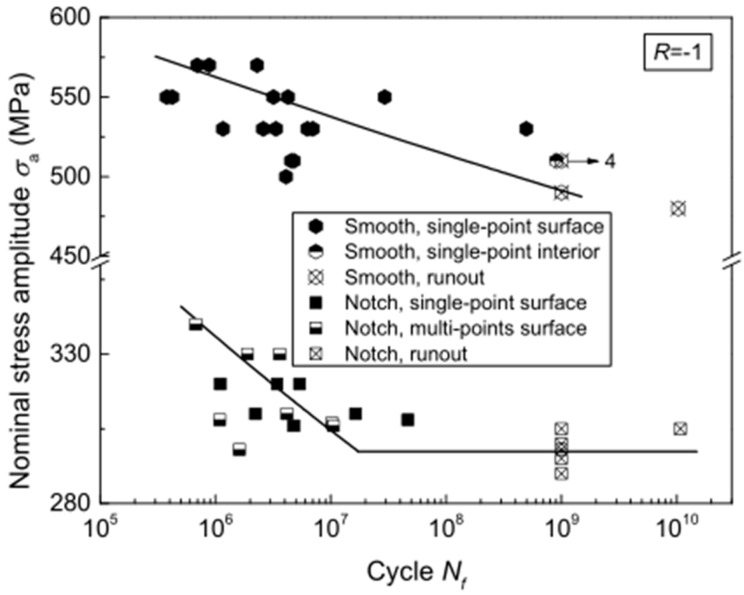
Comparison of fatigue data for a smooth specimen and a notched specimen for R = −1 [67].

In the same year, Gao et al. [66] used the ultrasonic method to study the effect of surface mechanical attrition treatment (SMAT) of a TC11 titanium alloy. They discovered that the SMAT reduced both the fatigue strength and the fatigue life of the alloy. Also, by using an ultrasonic fatigue testing method in 2018, Yang et al. [67] investigated the effect of notches on a near-α titanium alloy. They found that changes in the geometric shapes of the notches caused the fatigue S–N curve to shift from a “no plateau, no limit” to a “single plateau with limit” pattern (Figure 27).

#### 4.1.3. Influence of the Environmental Condition on the S–N Curve

**Figure 28:**
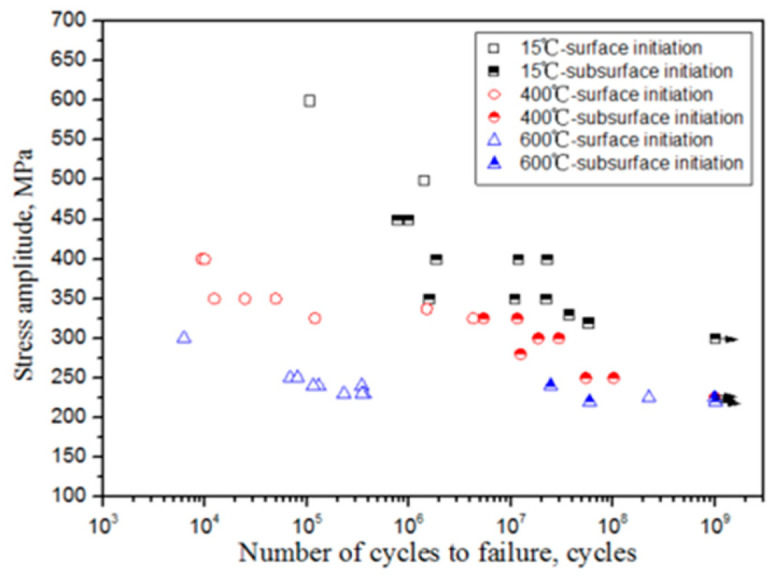
S–N curve of a TA29 titanium alloy at different temperatures [68].

The macroscopic characteristics of the S–N curve are also affected by the environmental condition. According to the public literature, researchers from various countries have mainly studied the effect of the environmental condition on the fatigue life of titanium alloy materials by considering the following two various aspects: working temperature and corrosive medium. In 2014, Zhao et al. [68] used the ultrasonic method to obtain S–N curves of the TA29 titanium alloy at the temperatures of 15 °C, 400 °C, and 600 °C. With an increasing temperature, the curve changed from a “no plateau, no limit” at 15 °C, through a “single plateau without limit” at 400 °C, and finally to a “single plateau with limit” at 600 °C (Figure 28).

In 2016, Li et al. [69] used both a rotating-bending method and an ultrasonic method to study the effect of a high temperature on the fatigue behavior of a Ti17 titanium alloy. All obtained S–N curves showed a “no plateau, no limit” pattern. At the temperatures of 200 °C and 350 °C, inflection points appeared in the S–N curve at approximately 10^7^ cycles, which was due to high-temperature oxidation.

**Figure 29:**
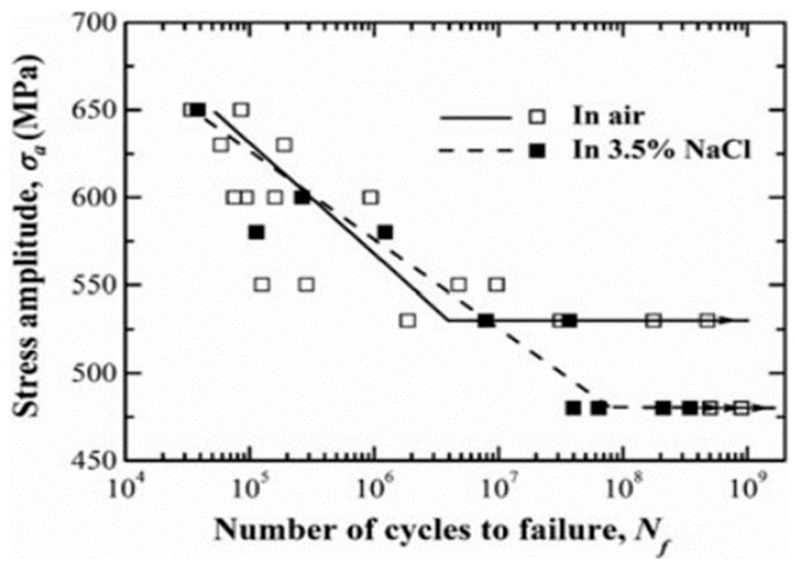
Fatigue life of Ti-6Al-4V in air and in a 3.5% NaCl solution [72].

Salt spray is the most common corrosive medium. In 2014, Dong et al. [70] performed vibration bending studies on the TC17 titanium alloy using the ultrasonic method. The S–N curve of the alloy exhibited a “no plateau, no limit” pattern. A slight reduction in the fatigue strength of the titanium alloy after corrosion was also found. The studies by Lu et al. [71] in 2016 also showed that the VHCF life of the specimens did not significantly decrease after corrosion. In 2017, Zhao et al. [72] studied the effect of a corrosive environment under rotating-bending loads on Ti-6Al-4V, and all obtained S–N curves showed a “single plateau with limit” pattern (Figure 29). Also, the titanium alloy exhibited a lower fatigue limit in a 3.5% NaCl solution.

Given the wide use of titanium alloys in medical equipment and artificial skeletons, the impacts of bodily fluids and alcohol on their fatigue strength have also been investigated. In 2011, Liu et al. [73] conducted an ultrasonic fatigue study on Ti-6Al-4V titanium alloy specimens after immersion in a simulated body fluid. As a result, their S–N curves showed “no plateau, no limit” patterns. Also, the VHCF life of the specimens decreased after immersion, but the duration of the immersion had a small impact on the life of the specimens. In 2015, Cao et al. [74] found that the S–N curve of TC4 titanium alloy, which had been treated with medical alcohol, exhibited a “single plateau with limit” pattern, whereas the S–N curve of the untreated TC4 titanium alloy showed a “no plateau, no limit” pattern.

### 4.2. Stress Ratio Influence and Goodman Diagram

Most aerospace components under actual operations do not bear symmetric loads, meaning that R > −1. For instance, the aircraft engine blades simultaneously persistently generate centrifugal forces and vibration stresses, with the stress ratio near the blade root typically larger than 0. In engineering, the Goodman diagram is often used to approximate the influence of the stress ratio. Experience shows that the Goodman diagram tends to exhibit a safety state within the HCF range. However, because of changes in the fatigue mechanisms, the patterns within the VHCF range require further investigations.

#### 4.2.1. Influence of Stress Ratio on the S–N Curve

**Figure 30:**
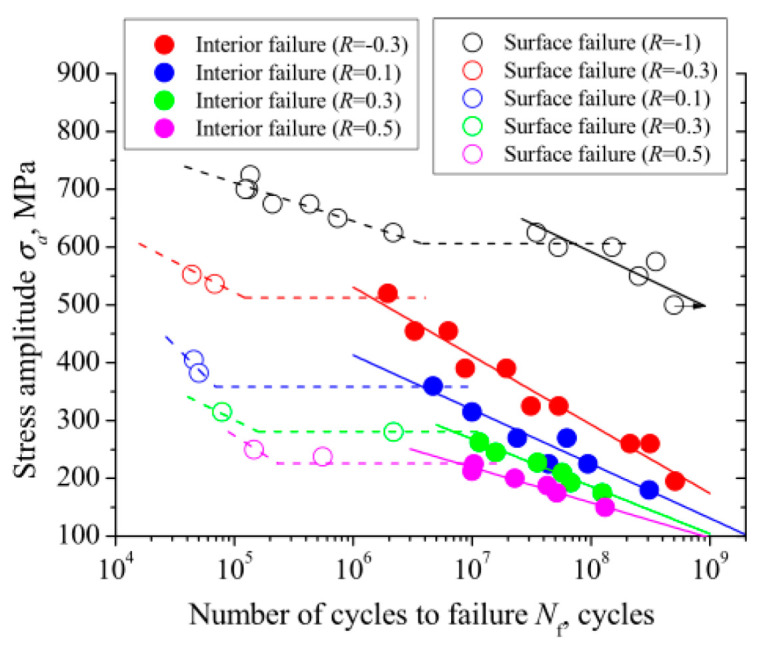
Relationship between stress amplitude and fatigue life for different stress ratios [31].

**Figure 31:**
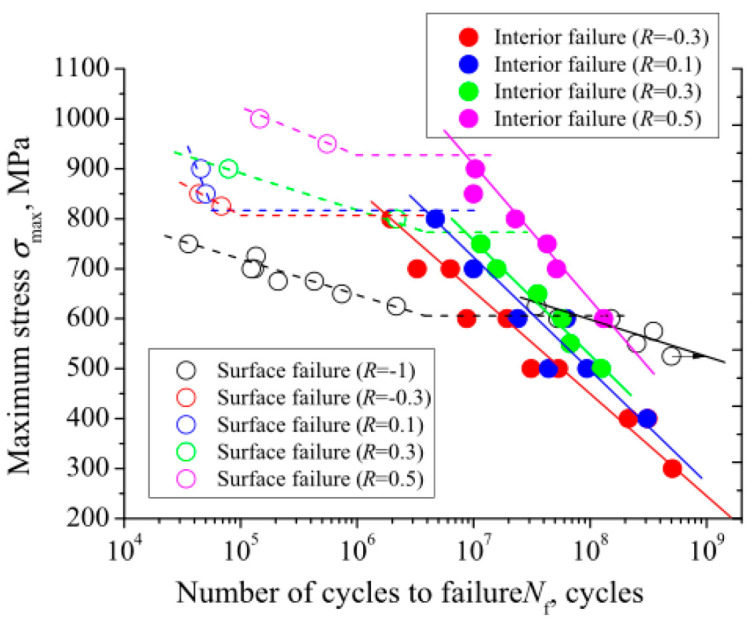
Relationship between maximum stress and fatigue life for different stress ratios [31].

By using a 100 Hz electromagnetic resonance testing machine, Li et al. [31] obtained, in 2017, S–N curves for TC4 titanium alloy under axial tension–compression at five different stress ratios. Figure 30 and Figure 31 present S–N curves for the stress amplitude and maximum stress, respectively, with all five stress ratios exhibiting a “single plateau without limit” pattern. However, as the stress ratio increases within the VHCF range, a transition takes place from a plateau region to a nonplateau region and from surface-initiated cracks to internally initiated cracks.

**Figure 32:**
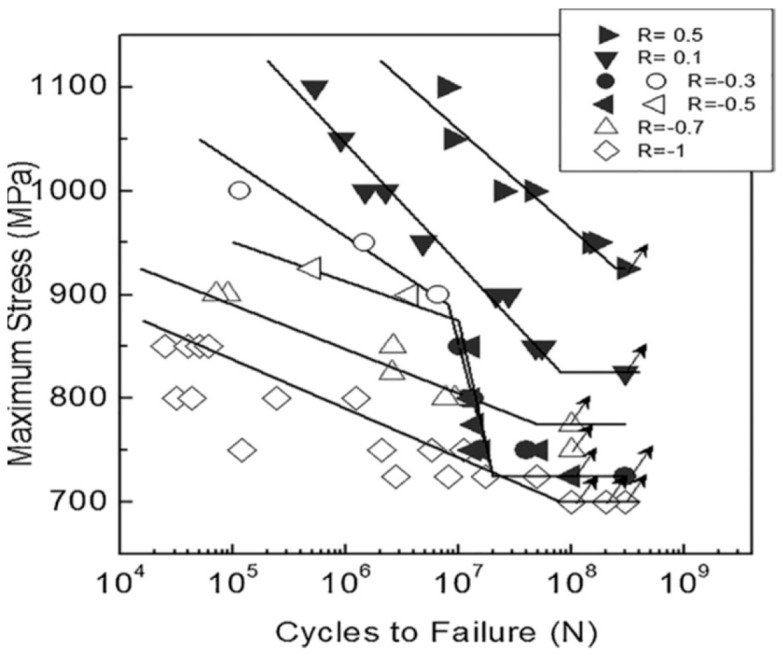
Stress amplitudes and fatigue life curves for six stress ratios [35].

In 2018, Wu et al. [35] used a 100 Hz electromagnetic resonance testing machine to obtain S–N curves for Ti-10V-2Fe-3Al titanium alloy under axial tension–compression at six different stress ratios (Figure 32). They demonstrate that the stress ratio significantly influences the form of the S–N curve.

**Figure 33:**
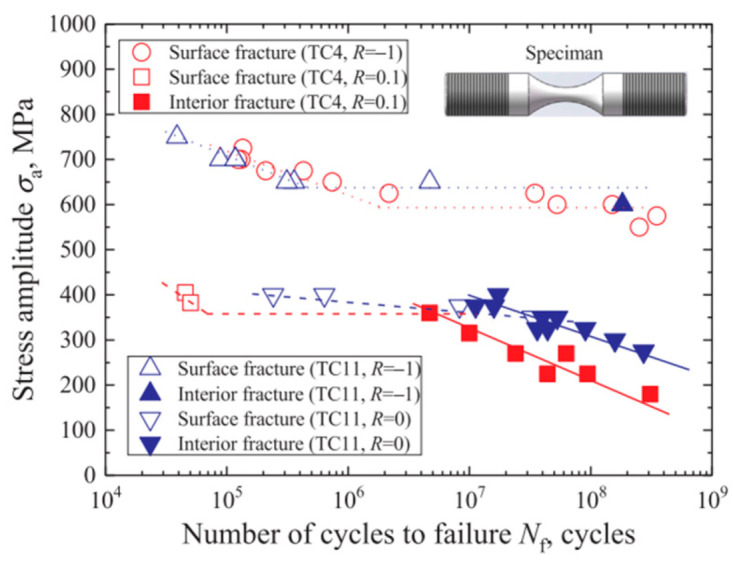
S–N curves for TC4 and TC11 titanium alloys at different stress ratios [34].

In 2020, Li et al. [34] used a 100 Hz electromagnetic resonance testing machine to compare the S–N curves for TC4 and TC11 titanium alloys under axial tension–compression at two different stress ratios (Figure 33). The results indicate that the stress ratio significantly influences the fatigue of TC4 and TC11 alloys. There were notable differences due to the specific grades of the titanium alloys. Compared to the compressive load, the tensile load had a more pronounced effect on reducing the fatigue strength.

**Figure 34:**
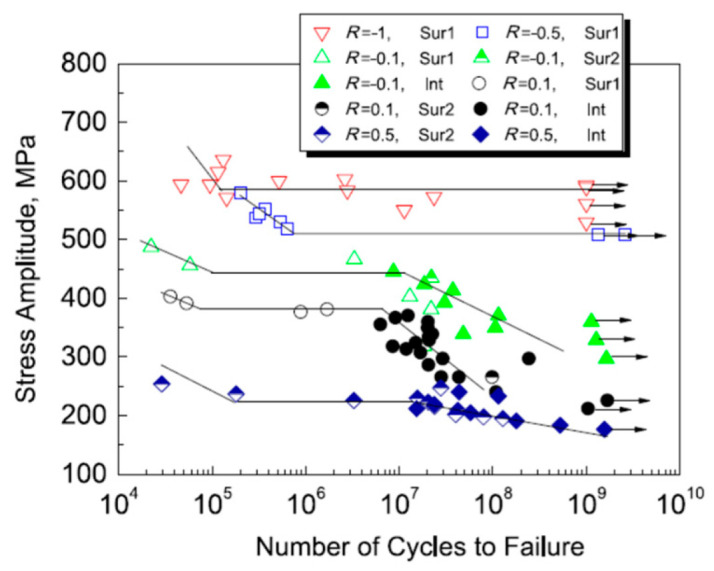
Relationship between stress amplitude and fatigue life [75].

**Figure 35:**
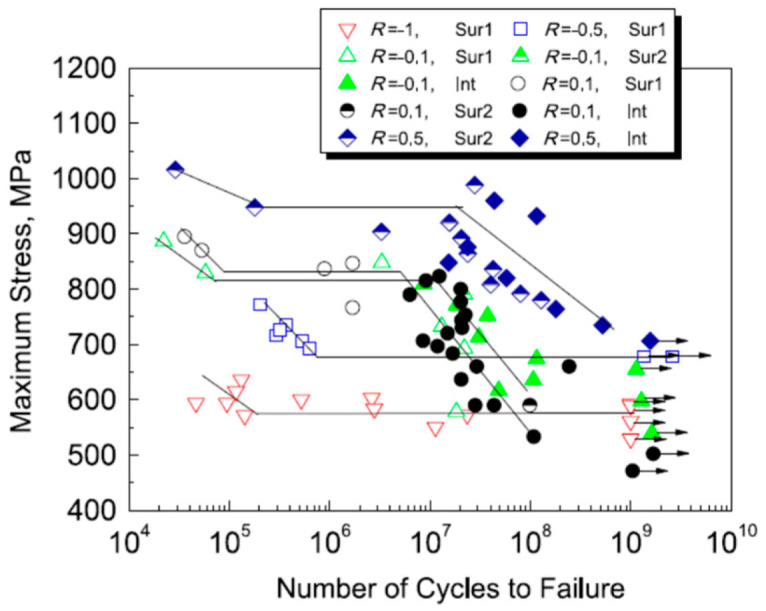
Relationship between maximum stress and fatigue life [75].

In 2017, Liu et al. [75] used a 20 kHz ultrasonic fatigue testing machine to obtain S–N curves for Ti-6Al-4V titanium alloy under axial tension–compression and for five different stress ratios. Under different stress conditions, Figure 34 and Figure 35 show the variations in the fatigue S–N curves for the stress amplitude and maximum stress, respectively. With the increase in stress ratio within the VHCF range, the profile shifted from a “single plateau without limit” to a “single plateau with limit”, and the crack initiation moved from the surface to the interior of the material.

**Figure 36:**
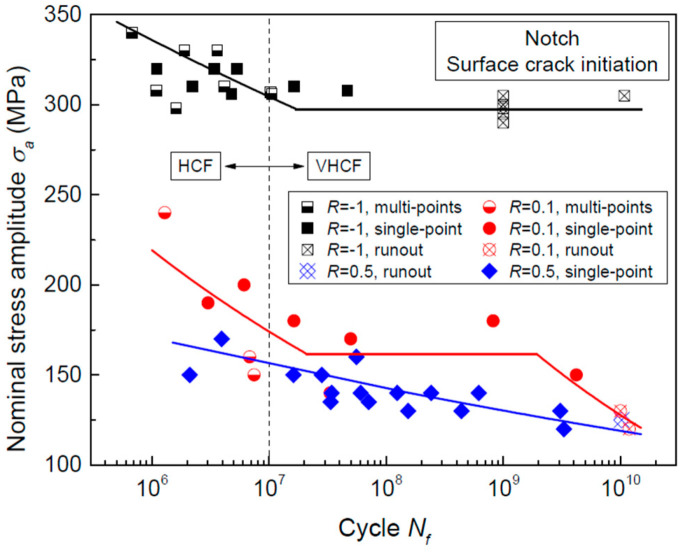
Ultrahigh-cycle fatigue life of Ti-8Al-1Mo-1 titanium alloy (with a notch) under axial tension and compression [67].

In the same year, Yang et al. [76] used a 20 kHz ultrasonic fatigue testing machine to obtain S–N curves for a Ti-8Al-1Mo-1V titanium alloy under axial tension–compression for three different stress ratios, all of which showed a “no plateau, no limit” profile. As the fatigue life increased, the trend of a decreasing stress amplitude remained fundamentally the same. However, the fatigue limit, which is represented by the maximum stress, significantly decreased, especially at higher stress ratios. As compared with the maximum stress, this showed that stress amplitude is a more reliable indicator for the impact of the stress ratio on the fatigue strength. In 2018, Yang et al. [67] used a 20 kHz ultrasonic fatigue testing machine to study the VHCF S–N curves of a notched Ti-8Al-1Mo-1V titanium alloy under axial tension–compression for three different stress ratios (Figure 36). The three S–N curves presented “single plateau with limit”, “single plateau without limit”, and “no plateau, no limit” patterns, respectively. These results show that the crack initiation mechanism underwent significant changes as the stress ratio increased.

#### 4.2.2. Goodman Diagram

**Figure 37:**
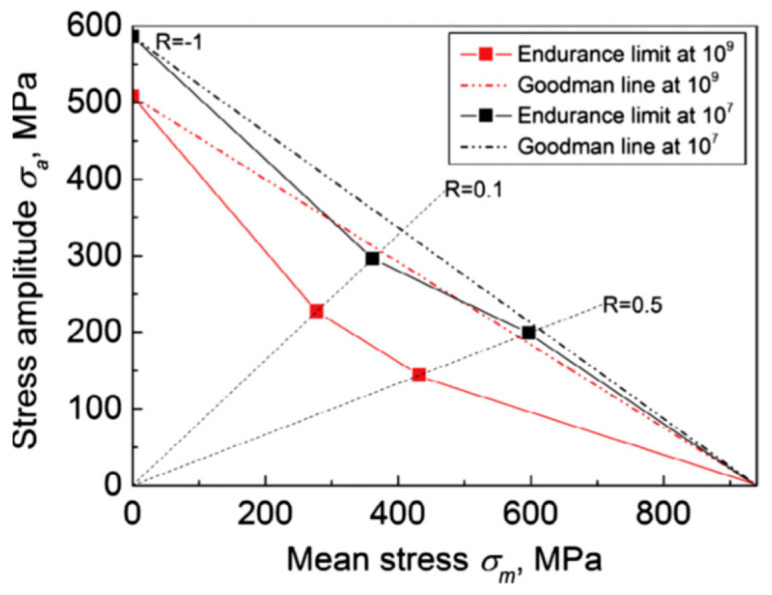
Goodman diagram presenting the ultrasonic fatigue data of Ti-8Al-1Mo-1V [76].

The Goodman diagram is typically considered to be a conventional estimate of the fatigue strength in the HCF domain. However, research studies have shown that the Goodman diagram provides an uncertain estimate of the fatigue strength of titanium alloys in the VHCF range. In 2017, Yang et al. [76] constructed a Goodman diagram based on ultrasonic fatigue test data of Ti-8Al-1Mo-1V within the VHCF range. As presented in Figure 37, the fatigue strength of Ti-8Al-1Mo-1V was found to be on the unsafe side in the Goodman diagram (at R = 0.1 and R = 0.5). Also, the deviation from the Goodman’s estimation was almost negligible at 10^7^ cycles. However, a significant discrepancy occurred at 10^9^ cycles. As compared with the HCF range, this was an indication of a more pronounced degradation of the fatigue limit in the VHCF range. Thus, the fatigue design should use a higher safety margin for R > −1.

**Figure 38:**
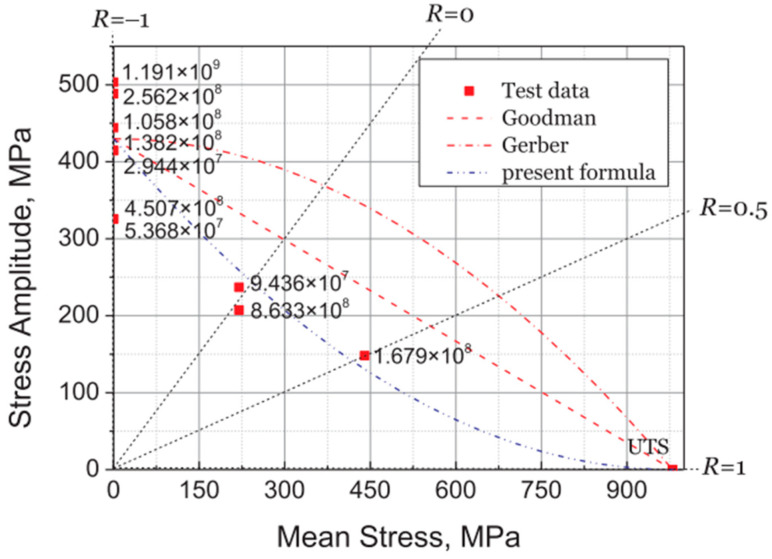
Analysis of a Goodman diagram for Ti-6Al-4V with ultrahigh cycle fatigue [77].

For the HCF and VHCF ranges, Pan et al. [77] constructed, in 2018, Goodman diagrams for Ti-6Al-4V, which were based on 20 kHz ultrasonic fatigue test data (Figure 38). The data deviated from the Goodman curve in the VHCF domain (i.e., R > −1), with the fatigue life falling into the estimated unsafe zone.

**Figure 39:**
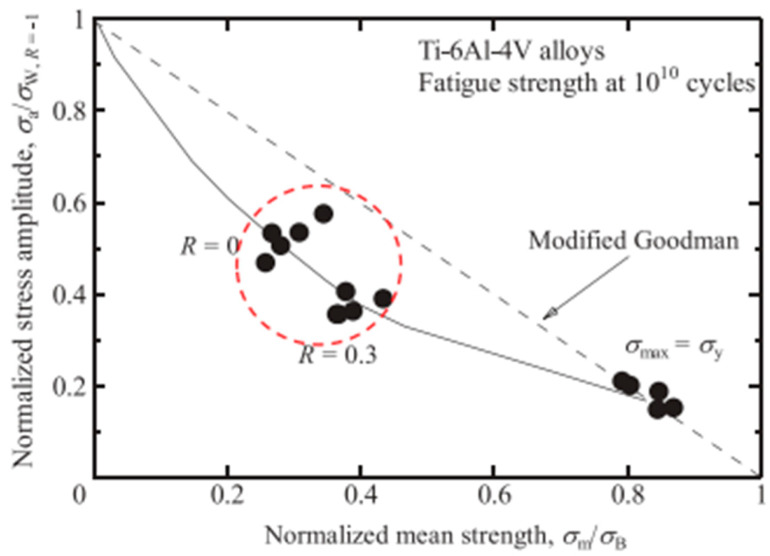
Analysis of the Goodman diagram of titanium alloy with ultrahigh cycle fatigue [22].

In 2019, Furuya et al. [22] plotted a Goodman diagram for Ti-6Al-4V in the VHCF range (Figure 39). Under high-stress-ratio conditions, the fatigue of Ti-6Al-4V titanium alloy was more likely to initiate in the interior of the material. This resulted in a substantial reduction in the VHCF strength, with the fatigue strength falling below the modified Goodman estimate.

### 4.3. Crack Initiation and Early Propagation Characteristics

For most titanium alloy materials that enter the VHCF stage, the crack initiation typically moves from the material surface to the subsurface. For high-strength steels, the internally initiated cracks often present a “fisheye” fracture morphology and are commonly referred to as the fine granular area (FGA). To maintain the consistency in the terminology, the present review has also adopted the concept of FGA for the characterization of internal crack initiation zones in the VHCF of titanium alloys. The shift in the mode of crack initiation inevitably leads to different fatigue fracture mechanisms. Therefore, the specific crack propagation characteristics require thorough investigations by the researchers.

#### 4.3.1. Fatigue Crack Propagation and Fundamental Feature Extraction

**Figure 40:**
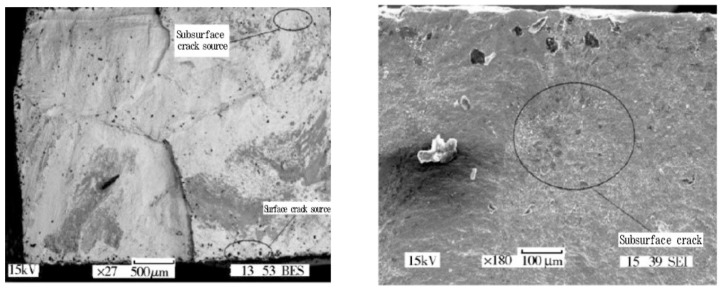
Analysis of the fracture morphology of TC17 titanium alloy using the ultrahigh-cycle bending vibration fatigue test [45].

In 1976, Neal et al. [78] studied the VHCF crack initiation mechanisms for Ti-4Al-4Mo-2Sn-0.5Si and Ti-6Al-4V under axial cyclic loading. They discovered that the fatigue cracks were primarily initiated in the interior of the materials, and were the first to show that the crack initiation stemmed from small cleavage planes that were formed by an internal α-phase cleavage. By using an ultrasonic bending fatigue test, Li et al. [45] found in 2012 that fatigue cracks could be initiated on both the surface and in the subsurface of the same specimen. These initiations were not depending on internal inclusions and defects, and a distinct “fisheye” damage morphology in the subsurface cracks was not observed (Figure 40).

**Figure 41:**
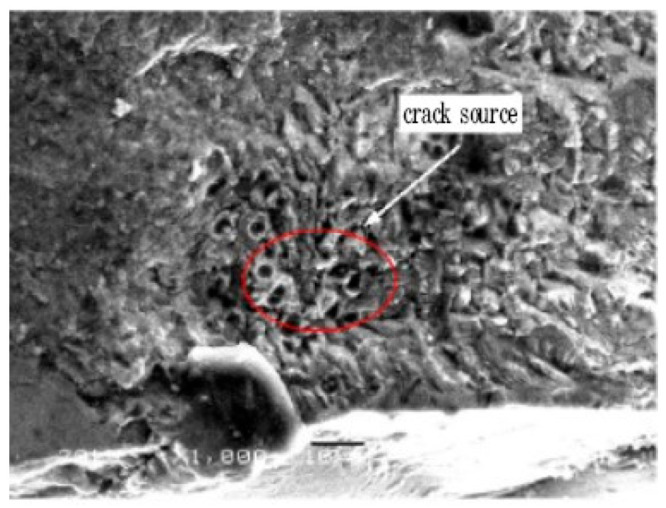
A spongy crack source in an ultrahigh-cycle fatigue titanium alloy [61].

In 2014, Huang et al. [79] investigated the VHCF behavior of TC17 titanium alloy (20 kHz, R = 0.1). They found that the fatigue cracks originated from the interior of the material. Small cleavage facets were observed in the crack initiation zone and intergranular fractures were clearly visible. When studying the VHCF characteristics of Ti6Al4V, Heinz et al. [80] discovered in 2016 a distinct FGA morphology in the initial areas of the fatigue cracks. They found cracks within the primary α-phase grains and were aligned with the FGA surface. In a study of the VHCF behavior of Ti-6Al-4V titanium alloy under bending vibration, Jiao et al. [61] observed in 2016 that the fatigue cracks were mainly initiated on the surface of the specimen, and a large number of quasi-cleavage steps were observed on the fracture surface. They used scanning electron microscopy (SEM) at a loading frequency of 300 Hz. In addition, at a loading frequency of 20 kHz, the fatigue cracks were initiated from either the surface or subsurface, with black sponge-like particles within the crack origin area (Figure 41).

**Figure 42:**
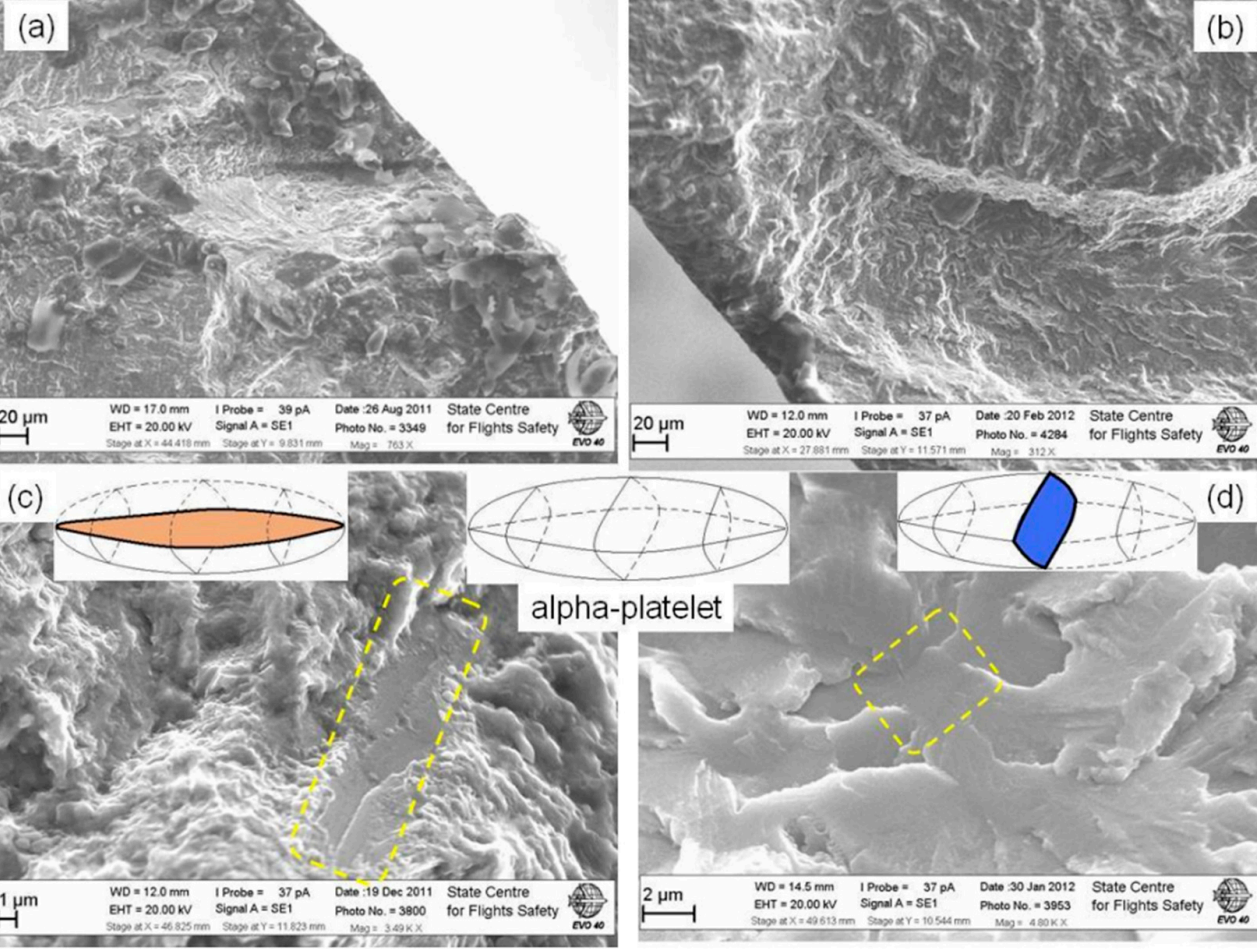
Forged VT3-1 crack initiation characteristics: (**a**) strong defect, (**b**) border of macro-zones, (**c**) quasi-smooth facet, and (**d**) smooth facet [60].

In 2016, Nikitina et al. [60] investigated the crack initiation mechanisms of the VHCF in a VT3-1 titanium alloy using a postforging process under tensile (R = 1 and R = 0.1) and torsional fatigue loadings. Fatigue cracks were then observed to initiate on both the surface and in the subsurface. For the subsurface crack initiation at a stress ratio of R = −1, several specific crack initiation behaviors were then identified: (1) initiation from strong defects, (2) initiation at the borders of “macro-zones”, (3) initiation from quasi-smooth facets, and (4) initiation from smooth facets. At a stress ratio of R = 0.1, the crack initiation was predominantly located at the borders of “macro-zones” (Figure 42).

**Figure 43:**
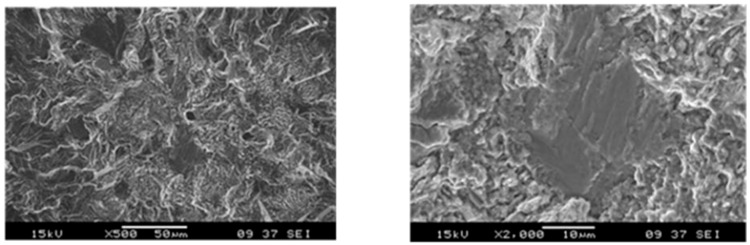
SEM morphologies of subsurface cracks in TC4 titanium alloy [42].

In 2017, Liu et al. [81] investigated the fatigue failure behavior of TC17 alloy for the loading frequencies of 110 Hz and 20 kHz. Regardless of frequency, the fatigue cracks in the material showed both surface and interior initiation, where the interior initiation was due to slip fracturing of the α phase. In 2018, Chen et al. [42] used a 20 kHz ultrasonic fatigue testing system to explore the VHCF characteristics of TC4 titanium alloy. Figure 43 shows that the cracks in the TC4 titanium alloy were initiated in the subsurface during the VHCF stage. They had a “quasi-fisheye” morphology, which differed from the traditional “fisheye” appearance.

**Figure 44:**
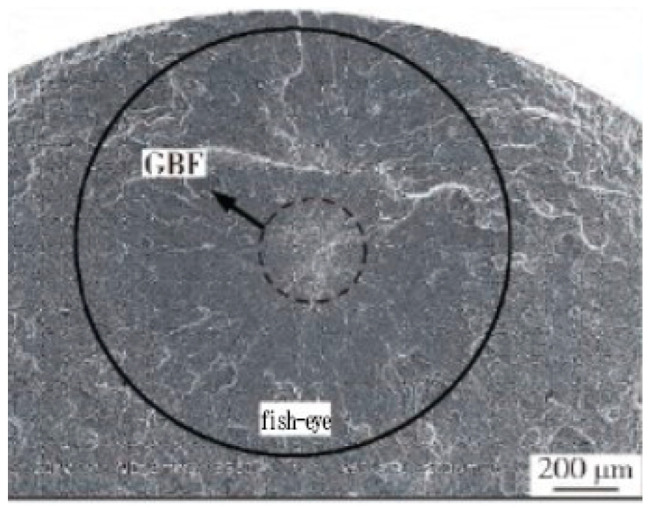
Crack initiation and fracture morphology on the surface and in the subsurface of TC4 titanium alloy (R = 0.1) [82].

In 2019, Gao et al. [82] performed VHCF tensile tests on TC4 alloy at different stress ratios (R = 0.1 and R = −1). The results showed that the cracks were initiated on the surface at a stress ratio of R = −1. For a stress ratio of R = 0.1, the fatigue crack initiation moved from the material surface to the interior as the stress decreased. Also, in the VHCF stage with a fatigue life of N > 10^7^, the fatigue cracks originated from the interior of the material and exhibited a typical “fisheye” morphology (Figure 44).

#### 4.3.2. Influence of the Specimen Condition on the Fatigue Crack Propagation

**Figure 45:**
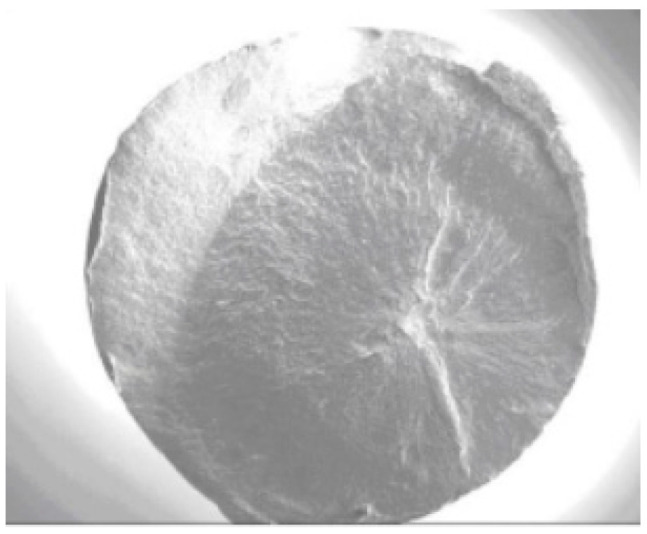
Fatigue fracture morphology of extruded VT3-1 ultrahigh cycle titanium alloy [84].

In 2011, Tian et al. [83] used an ultrasonic fatigue testing method to study the effect of plasma nitriding treatment on the fatigue properties of titanium alloys. They discovered that crack initiation primarily occurred on the surface and in the subsurface of the specimens for N < 10^8^. In addition, inclusions significantly affected the locations of the crack initiations. In 2014, Nikitin et al. [84] examined the VHCF crack initiation mechanisms of α–β titanium alloys treated with the following two different processes: forging and extrusion. For a stress ratio of R = −1, the cracks in the extruded titanium alloy were initiated in the interior of the material. On the other hand, in the forged titanium alloy, the crack initiation moved from the surface to the subsurface within a fatigue life range of 10^6^–10^7^. In addition, the fracture morphology of the extruded titanium alloy resembled that commonly seen in high-strength steels, and “butterfly wings” were observed on the fracture surfaces of the extruded VT3-1 titanium alloys (Figure 45). At a stress ratio of R = 0.1, the fatigue cracks in the forged titanium alloy were initiated on both the surface and in the subsurface, while the fatigue cracks in the extruded titanium alloy were located in the interior of the alloy.

**Figure 46:**
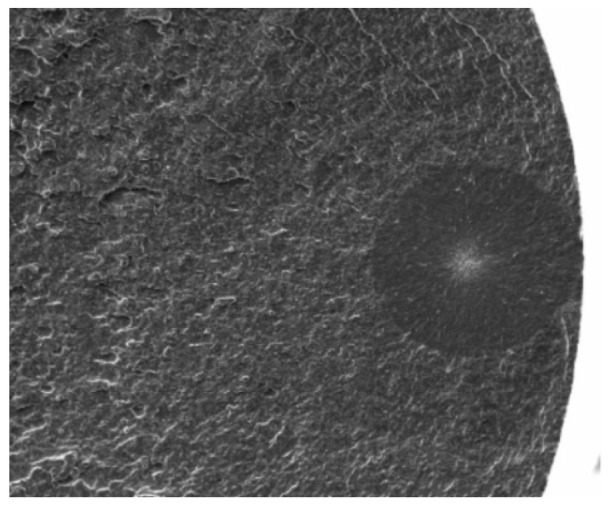
Fracture morphology of subsurface crack initiation in a TC11 specimen [66].

Using a 20 kHz ultrasonic testing device and a stress ratio of R = −1, Shoichi et al. [85] performed, in 2014, a detailed study on the impact of the low-temperature nitriding process on the VHCF properties of Ti-6Al-4V alloy. The study revealed that as the fatigue life increased, the failure mode of the specimens shifted from a surface failure to a subsurface fatigue failure. A typical “fisheye” morphology was then observed. In 2016, Zhu et al. [65] investigated the impact of the surface roughness on the VHCF properties of Ti-6Al-4V alloy specimens. They found that with an increase in surface roughness, the VHCF cracks tended to initiate from the interior toward the subsurface, and the source of the fatigue cracks changed from a single source to multiple sources. When studying the effect of the SMAT on the VHCF properties of a TC11 titanium alloy (20 kHz, R = −1), Gao et al. [66] found, in 2018, that untreated specimens showed both surface and interior crack initiation modes, with the interior crack initiation occurring after 10^8^ cycles. In addition, a distinct “fisheye” structure was observed in the interior crack initiation zone (Figure 46), and it was assumed that the interior crack initiation was caused by the heterogeneity of the microstructure. Also, specimens that had been treated with SMAT presented crack initiations solely from the specimen surface.

**Figure 47:**
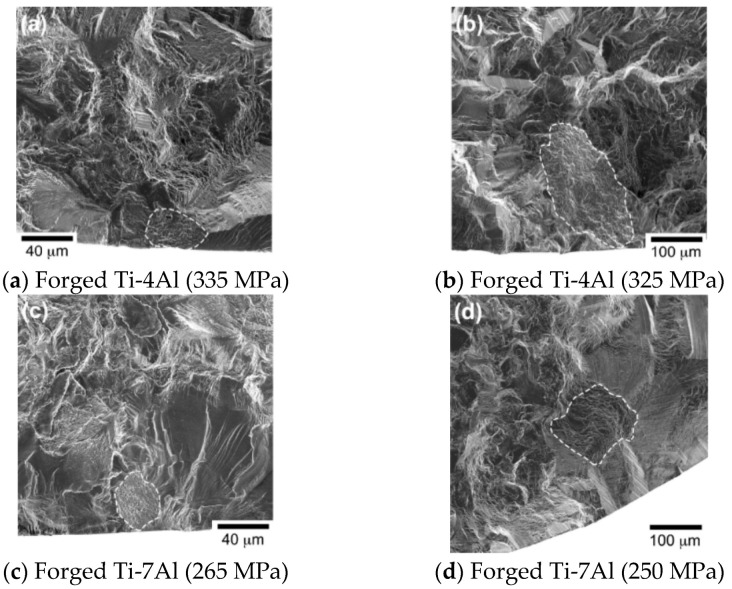
Morphologies of ultrahigh-cycle fatigue fractures for different stress levels and alloys [86]: (**a**) 335 MPa for forged Ti-4Al; (**b**) 325 MPa for forged Ti-4Al; (**c**) 265 MPa for forged Ti-7Al; (**d**) 250 MPa for forged Ti-7Al specimens.

Using an ultrasonic fatigue system, in 2019, Shi et al. [86] investigated systematically the HCF and VHCF properties of three binary Ti-Al alloys with a single α phase (R = −1). Regions mixed with tearing topographical surface (TTS) features and small cleavage facets were then observed on the fatigue fracture surfaces (Figure 47).

#### 4.3.3. Influence of Environmental Condition on Fatigue Crack Propagation

**Figure 48:**
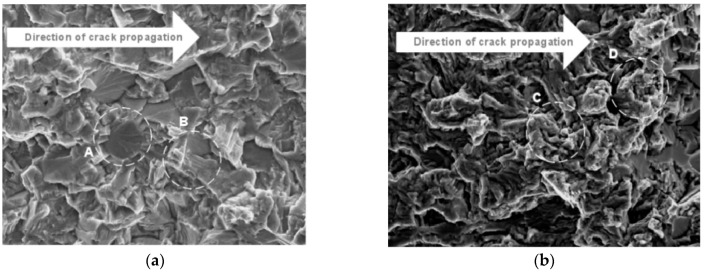
Surfaces of crack propagation fractures on a titanium alloy in different environments: (**a**) air; (**b**) HV [24].

In 2010, Oguma et al. [24] studied the evolution characteristics of the fatigue crack damage of Ti-6Al-4V alloy in a vacuum environment. The surface of the fatigue fracture showed clear cleavage facets, serrated crystals, and slip traces in air and medium vacuum (MV) environments. On the other hand, pronounced granular feature areas were observed on the fracture surfaces in high vacuum (HV) and ultrahigh vacuum (UHV) environments (Figure 48).

**Figure 49:**
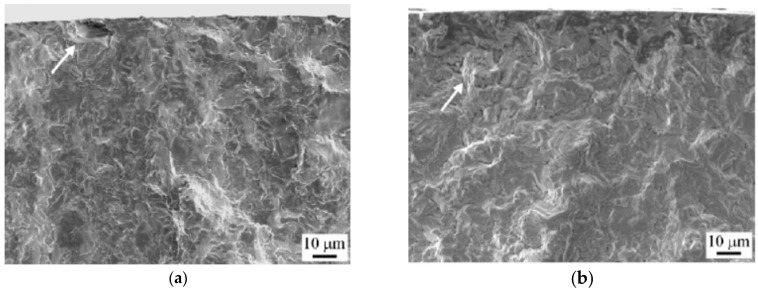
Morphologies of ultrasonic fatigue fractures on a TC17 specimen at room temperature and for various grades: (**a**) 600 MPa; (**b**) 560 MPa [87].

In 2014, Li et al. [87] investigated the VHCF characteristics of TC17 titanium alloy under high-temperature conditions. They found that cracks typically initiated in the subsurface region at room temperature and under low-stress conditions. At 350 °C, cracks initiated on the surface of the specimen at different stress levels, with a significant amount of white flocculent material at the origin of the crack (Figure 49).

In 2014, Zhao et al. [68] studied the VHCF performance of an aeronautical engine integral compressor disk at temperatures of 15 °C, 400 °C, and 600 °C (20 kHz, R = −1). The disk was made of TA29 titanium alloy. At 15 °C, most of the fatigue cracks initiated from the subsurface or interior of the material, with cleavage facets comparable in size to α-phase grains. At the temperatures of 400 °C and 600 °C, finer and rougher microstructures, respectively, were observed in the crack initiation area. In 2016, Lu et al. [71] investigated the effect of salt spray corrosion on the VHCF of Ti-6Al-4V. A comparison of fatigue fracture surfaces of corroded and uncorroded specimens revealed that the fatigue cracks in both types of specimens were initiated on the surfaces when the fatigue life was below 10^8^ cycles. For a fatigue life longer than 10^8^ cycles, the cracks in the corroded specimen were still initiated on the surface, while the fatigue cracks in the uncorroded specimen originated from the interior of the material. In 2017, Jiao et al. [88] studied the VHCF behavior of the TA11 titanium alloy under high-temperature conditions. They discovered that the mode of crack initiation was influenced by the stress ratio at high temperatures. Fatigue cracks were initiated on the surface for the stress ratios R = −1 and R = 0.1. Furthermore, cracks were initiated in the interior of the material, with a typical “fisheye” morphology, at the stress ratio R = 0.5. In addition, cleavage facets appeared in the crack initiation zone.

## 5. Model Construction of the VHCF

Because of limitations related to the experimental analysis techniques and the complexity of the theoretical analysis, the current research on the VHCF of titanium alloys have primarily focused on phenomenological analyses based on experimental results. Just a few models have then been used to study the fatigue fracture mechanisms. Although it is possible to intuitively reconstruct the complete physical process of fatigue fracturing in titanium alloy materials with phenomenological analyses, it is essential to delve into existing experimental results and construct analytical models that are applicable to VHCF fracturing of titanium alloys. It would, thereby, be possible to further deepen the theoretical understanding and improve the level of real component design. This section elaborates on three main categories of fatigue fracture model analyses for titanium alloy materials, which are based on the following different emphases: (1) model analysis of the fracturing process from a material science perspective; (2) analysis of the characteristic parameters from a fracturing mechanics perspective; and (3) simulation and finite element numerical analyses from a computational mechanics perspective.

### 5.1. Crack Initiation Mechanisms and Models

Cracks in the VHCF typically originate from the interior of the material. To grasp the unique behavior of fatigue fracturing and predict the fatigue life in the VHCF, it is crucial to fully understand the behavior of the fatigue fracturing in titanium alloys. It is also crucial to comprehend the mechanisms of crack initiation and unveil the formation mechanism of the FGA in the crack initiation zone. Over the past decade, many scientists have explained the formation mechanism and presented models of the VHCF crack initiation zone from various material science perspectives, including the following mainstream ideas (see below).

#### 5.1.1. Grain Dislocation and Slip Motion Model Based on Fatigue Loading

**Figure 50:**
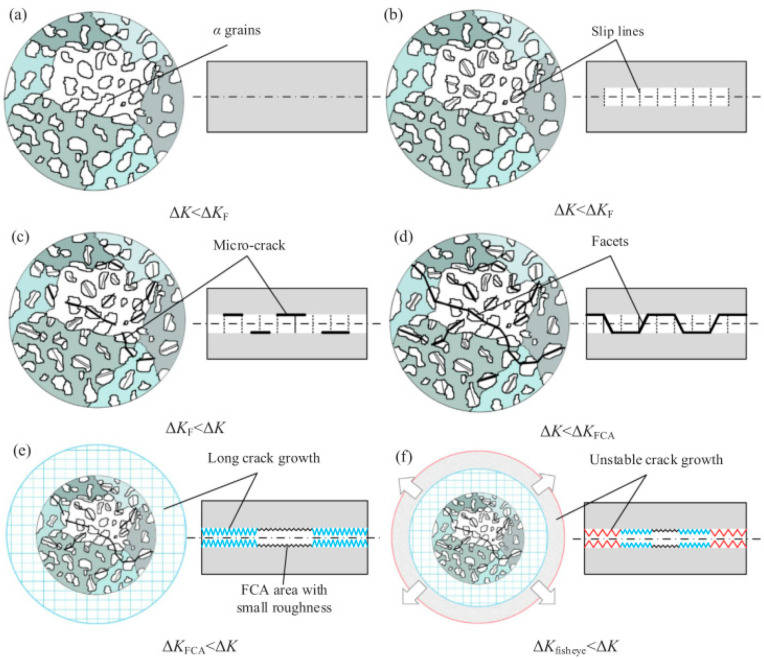
Diagram of crack evolution during ultrahigh cycle fatigue [33].

Li Wei et al. [31,33] and Liu X et al. [89] believe that the principle of fatigue failure in titanium alloys can be understood from the following scenario: Isolated primary α grains undergo cleavages under the continuous action of fatigue loads, thereby forming a series of slip lines within the specimen. However, in this scenario, the ΔK in the matrix remains smaller than the crack propagation threshold, ΔK_F_. As the fatigue process progresses, more slip lines, or bands, become the sources of cracks, thereby forming several discontinuous microcracks within the α grains. With a continuous increase in the number of cycles, microcracks will coalesce and propagate, in which case the ΔK at the crack tip will exceed ΔK_F_. When microcrack initiation, coalescence, and propagation lead to fracturing of the α grains, a faceted planar crack (i.e., FCA) will form. As the crack size increases and the ΔK exceeds ΔK_FCA_, adjacent microcracks will form a macroscopic crack and propagate at a stable growth rate. When the “fisheye” morphology is fully formed, the subsequent crack will propagate in an unstable manner until an instantaneous fracturing occurs (Figure 50).

#### 5.1.2. Crack Propagation Model Based on Grain Orientation

**Figure 51:**
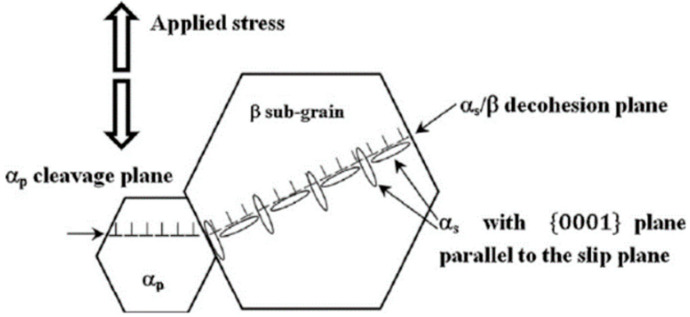
Schematic diagram of crack propagation with different cleavage mechanisms [35].

In 2014, Everaerts et al. [90] used SEM to explore the fatigue fracture mechanism of Ti-6Al-4V under tensile conditions. They discovered that the crack initiation tended to move from the surface toward the interior of the material in the HCF regime. Additionally, based on the crystalline structure of the material and the spatial distribution of the facet angles, the angle between the facet and the normal was relatively large. The fatigue cleavage facets were most likely aligned with the prismatic lattice planes. In 2018, Wu et al. [35] studied the characteristics of the VHCF crack propagation in dual-phase Ti-10V-2Fe-3Al titanium alloy. They identified an $\alpha_{p}$-phase cleavage and $\alpha_{s}$/β-phase cleavage as two different crack initiation mechanisms. In addition, they noted that the microstructural neighborhood might compete, thereby leading to the initiation of the main crack. The stress ratio directly determined the outcome of the competition between the two crack initiation mechanisms (Figure 51).

#### 5.1.3. Crack Propagation Model Based on the Torsional Damage Mechanism

**Figure 52:**
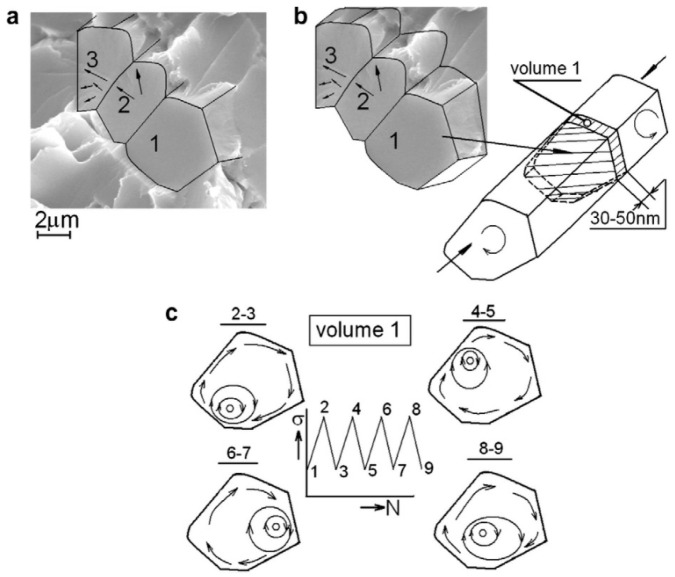
Schematics of the crack evolution mode based on the torsional damage mechanism [91].

Using the acoustic emission (AE) effect, Shanyavskiy et al. [91] studied, in 2010, the initiation and expansion morphologies of fatigue cracks by monitoring signal changes during the fatigue processes in the specimens. The propagation paths of the short cracks were predominantly characterized by rotational or torsional modes (Figure 52). A smooth crack initiation surface formed during the damage accumulation period. This crack propagation pattern was closely related to the trajectory of the fatigue stress loading, as accompanied by plastic deformations inside the specimen, the internal residual stresses dominated the local deformation mode of the material.

#### 5.1.4. Thermal Analysis Based on the Fatigue Damaging Process

In 2014, Huang et al. [92] reconstructed a model of the temperature variation within a thermodynamic framework by estimating the anelastic and inelastic thermal dissipations at the micro-active sites within the reference volume of the element. The method of failure probability prediction was used to investigate the changes in temperature during the fatigue loading process. The accuracy of the model was subsequently confirmed by experimental validation.

### 5.2. Analyses of the Characteristic Parameters for Crack Initiation and Initial Propagation

Under fatigue loading, titanium alloy materials must inevitably go through stages of crack initiation and propagation before experiencing fatigue failure. When a fatigue crack reaches a certain size and the stress intensity factor exceeds the threshold for fatigue crack propagation, it leads to a rapid expansion of the fatigue crack, which ultimately causes a fracture in the specimen. From the existing literature, analyses of the characteristic parameters for titanium alloy fatigue fracturing are primarily based on the traditional fracturing mechanics of crack initiation, propagation of stress intensity factors, and their variations (including the crack size ($\sqrt{area}$) model, D-factor model, and crack length analysis).

#### 5.2.1. Analysis of the Crack Size Model

**Figure 53:**
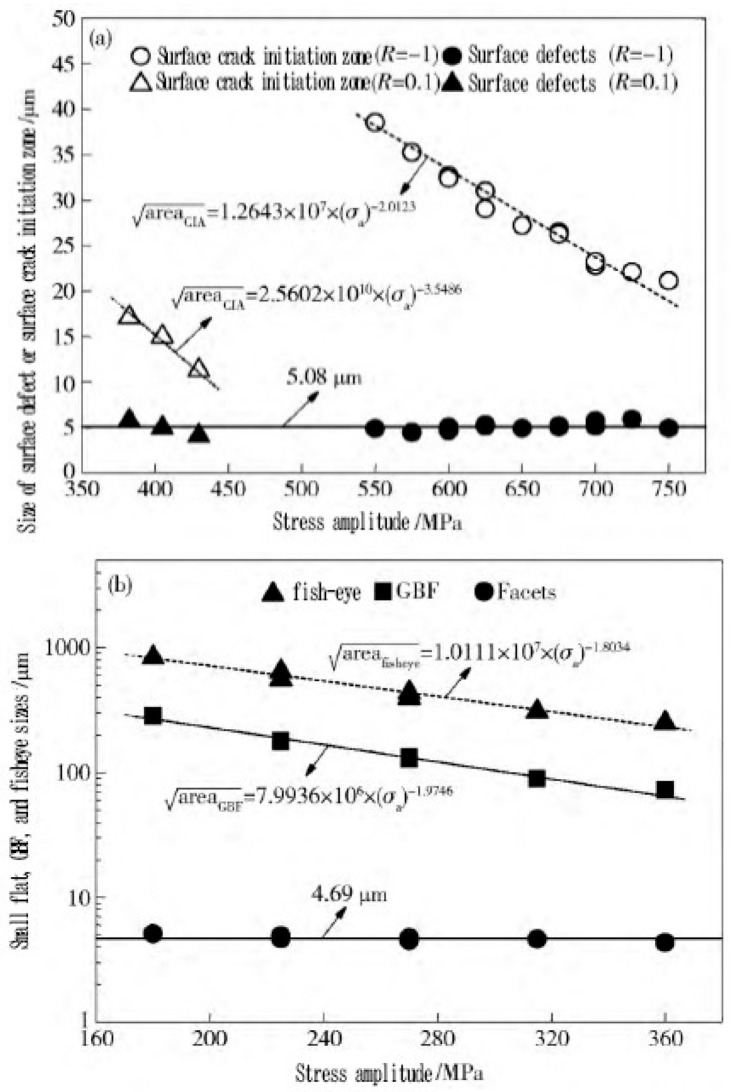
Relationship between the defect, or crack size, and the stress amplitude: (**a**) surface failure; (**b**) internal failure [82].

In 2019, Gao et al. [82] studied the VHCF characteristics of TC4 titanium alloy using an electromagnetic resonance fatigue testing machine. In addition, they used the $\sqrt{area}$ parameter to analyze the failure mechanism in the TC4 titanium alloy for different stress ratios. As can be seen in Figure 53, the stress ratio significantly affected the fatigue crack propagation characteristics of the surface crack initiation zone. Furthermore, the value of $\sqrt{area}$ was found to be independent of the stress ratio and stress amplitude for the surface defects. For R = 0.1, the value of $\sqrt{area_{facet}}$ was independent of the magnitude of the stress amplitude, while $\sqrt{area_{GBF}}$ and $\sqrt{area_{fisheye}}$ decreased with an increasing stress amplitude.

#### 5.2.2. Analyses of the Damage Threshold and Fatigue Life Characteristics Based on the Crack Growth Rate

**Figure 54:**
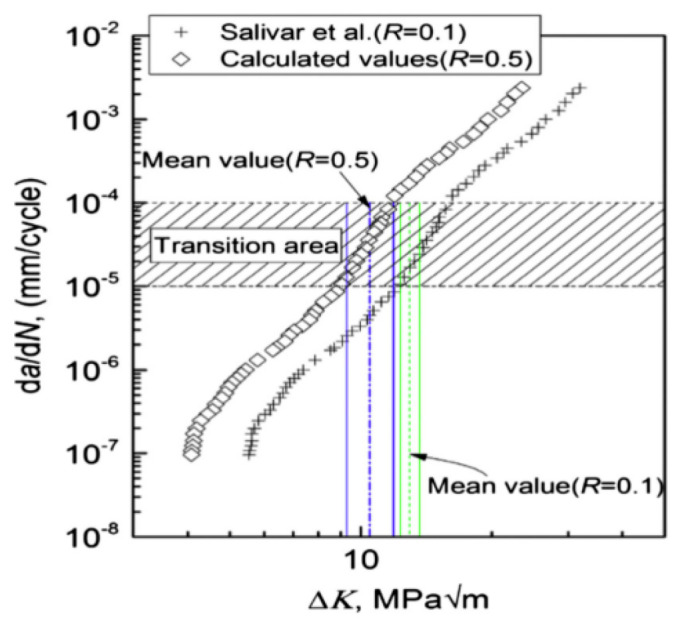
Analysis of the crack growth rate for different stress ratios [76].

Using ultrasonic fatigue testing, Yang et al. [76] found, in 2017, that the Paris law accurately represents the crack growth rate in the VHCF regime of Ti-8Al-1Mo-1V titanium alloy (Figure 54). The variation in the damage threshold aligned well with the characteristics of the fatigue fracture surface.

#### 5.2.3. Analysis of the Crack Competition Mechanism

**Figure 55:**
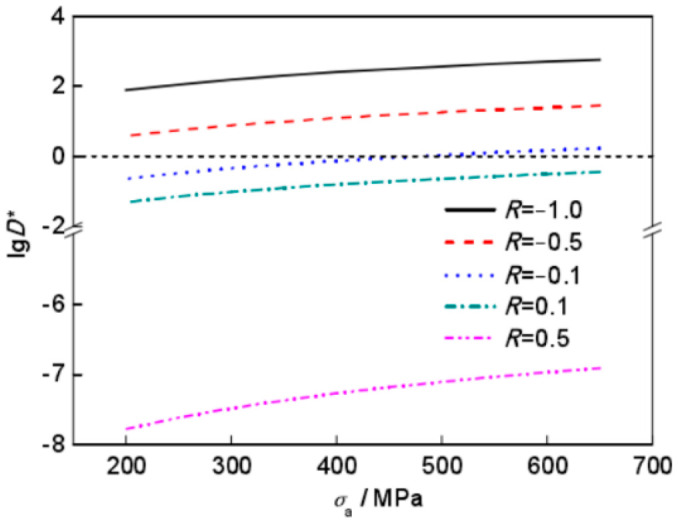
Relationship between $\mathrm{lg}D^{*}$ and stress amplitude for different stress ratios [75].

Cracks will generally initiate in the subsurface and in the interior of titanium alloys that undergo VHCF. However, under certain conditions, cracks in some titanium alloys may even initiate on the surface. In 2016, Liu et al. [75] noted that the VHCF failure in the Ti-6Al-4V alloy involves both slip and cleavage mechanisms. For these situations, the introduction of the parameter D* serves to represent the competition between surface and internal crack initiations. As presented in Figure 55, the crack initiation mechanism is closely related to the stress ratio and the stress amplitude.

### 5.3. Numerical Analysis Models

#### 5.3.1. Physical Fatigue S–N Model

**Figure 56:**
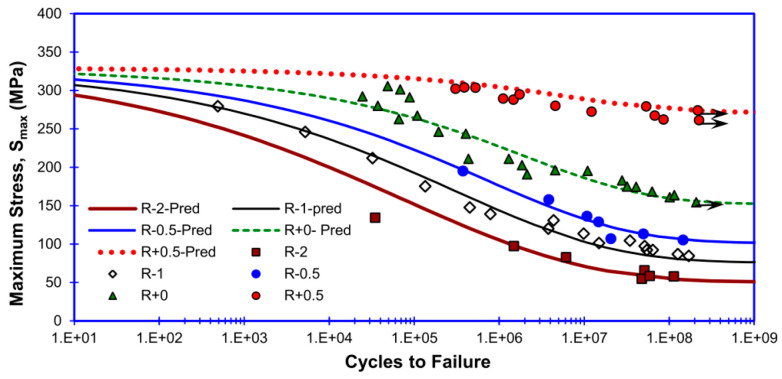
Predicted results based on the fatigue life model [93].

By postulating that physical endurance limits the stress condition corresponds to the critical shear stress for a slip. Chandran et al. [93] integrated, in 2016, the fatigue crack growth function with physical boundary conditions in the development of a constitutive equation. This equation was found to be suitable for the prediction of the fatigue lives of both single-crystalline and polycrystalline materials. Furthermore, it could be extended to include the effects of the average stress in various forms, thereby enabling a complete prediction of the S–N behavior and fatigue limits under any average stress. The resulting equation was solely based on the S–N behavior with respect to fully reversed fatigue data (Figure 56).

#### 5.3.2. Analysis Model of the Damage Evolution

In 2011, Przybyla et al. [94] used correlation functions to link the response parameters of the extreme value fatigue with microstructural attributes at critical fatigue sites. They compared the stress characteristics of fatigue crack nucleation with early expansion under room temperature conditions for four different microstructural variants of Ti-6Al-4V. The simulation results showed that microstructures with smaller crystalline sizes and volume fractions of primary α grains tended to show less variability in and lower magnitude of the stress required for fatigue crack formation.

## 6. Conclusions

In the present work, the latest developments in the VHCF research on titanium alloys were systematically reviewed and summarized from 2010 to date. This was performed with a special focus on (i) experimental methods, (ii) macroscopic and microscopic characteristics of fatigue fractures, and (iii) construction of fatigue fracture models. The purpose of the present review is to acquire a deeper understanding about the VHCF behavior of titanium alloys.

### 6.1. Research Techniques, Crack Propagation, and Analysis Models for VHCF Issues in Titanium Alloys

(1) This is a systematic review of the experimental methods and in situ testing techniques that have been used in the research on the VHCF properties of titanium alloys. Ultrasonic methods have mostly been used, followed by hydraulic servo and electromagnetic resonance methods. No significant frequency effect can be observed in the VHCF regime. However, new loading methods continue to be developed.
(2) This review provides a detailed introduction to the characteristics of the S–N curves and Goodman diagrams for titanium alloys with VHCF, as well as the crack initiation mechanisms. There are four types of S–N curves for titanium alloys, which are largely influenced by the material’s condition, loading mode, and working environment. With an increasing fatigue life, the Goodman diagrams become increasingly unsafe at R > 0. In addition, crack initiation increasingly takes place within the material.
(3) This review organized existing analyses of the characteristic parameters for crack initiation and initial propagation in the VHCF of titanium alloys in a comprehensive way. In the fracturing process for titanium alloys, the analyses dissected the crack development and variation in the characteristic parameters from the following three perspectives: material mechanics, fracture mechanics, and computational mechanics.
(4) This review provides a methodical summary of the predictive models of the VHCF life of titanium alloys. Such models are based on constitutive models of the materials and can, to a certain extent, accurately reflect the basic mechanical properties of the materials and the influence of external loads on the VHCF life.

### 6.2. Areas Requiring Further Research on the VHCF of Titanium Alloy Materials

(1) This review presents models of early damage initiation and propagation for the VHCF in titanium alloys, which are based on physical mechanisms. The current models for early damage and propagation are mostly derived from the “fisheye” model for high-strength steels. However, titanium alloys do not generally contain nonmetallic inclusions, which raises significant doubts about the applicability of the “fisheye” model for titanium alloys.
(2) This is a review of high-precision online detection techniques for analyses of the initiation and propagation processes of VHCF damage. The study of fatigue crack evolution mechanisms requires advanced experimental instruments and cutting-edge detection technology, especially for the damage evolution characteristics of the FGA fatigue crack source zone at the micrometer scale. Furthermore, to advance the translation of scientific research into engineering achievements, it is essential to develop online detection technologies for external environmental conditions.
(3) This review includes the transition from fatigue properties of titanium alloy materials to engineering structure applications. Current research focuses primarily on the fatigue properties of materials. The performance of fatigue experiments under environmental conditions, for various loads and surface properties, and with cumulative damage rules for high/low-cycle fatigue loads, is a major issue for future VHCF research on titanium alloys.
(4) The present review includes numerical simulation models and methods for VHCF in titanium alloys. The establishment of numerical models that quantitatively describe the damage behavior and predict the performance of VHCF is crucial for the systematic construction of a theoretical framework for VHCF.

## Data Availability

The raw data supporting the conclusions of this article will be made available by the authors on request.
